# A novel framework with automated horizontal pleiotropy adjustment in mendelian randomization

**DOI:** 10.1016/j.xhgg.2024.100339

**Published:** 2024-08-02

**Authors:** Zhaotong Lin

**Affiliations:** 1 Department of Statistics, Florida State University, Tallahassee, FL, USA; 2 Division of Biostatistics and Health Data Science, School of Public Health, University of Minnesota, Minneapolis, MN, USA

**Keywords:** biological pleiotropy, conditional genetic effect, LD-induced pleiotropy, two-stage framework

## Abstract

The presence of horizontal pleiotropy in Mendelian randomization (MR) analysis has long been a concern due to its potential to induce substantial bias. In recent years, many robust MR methods have been proposed to address this by relaxing the “no horizontal pleiotropy” assumption. Here, we propose a novel two-stage framework called CMR, which integrates a conditional analysis of multiple genetic variants to remove pleiotropy induced by linkage disequilibrium, followed by the application of robust MR methods to model the conditional genetic effect estimates. We demonstrate how the conditional analysis can reduce horizontal pleiotropy and improve the performance of existing MR methods. Extensive simulation studies covering a wide range of scenarios of horizontal pleiotropy showcased the superior performance of the proposed CMR framework over the standard MR framework in which marginal genetic effects are modeled. Moreover, the application of CMR in a negative control outcome analysis and investigation into the causal role of body mass index across various diseases highlighted its potential to deliver more reliable results in real-world applications.

## Teaser

Mendelian randomization can be used to infer the causal effect of exposure on outcome, but results are often biased by horizontal pleiotropy. Lin et al. present a universal MR framework called CMR to automatically adjust for pleiotropy, which can improve the performance of many existing MR methods.

## Introduction

**Figure 1:**
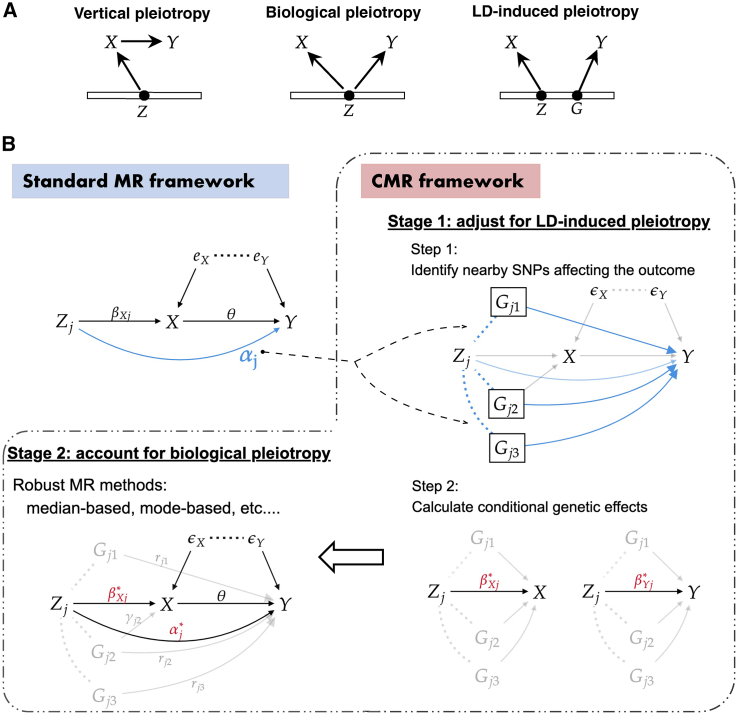
Conceptual framework of the study (A) Three types of pleiotropy happen in MR. *Z* is the genetic instrument, *X* is the exposure, *Y* is the outcome, and *G* is an SNP in LD with *Z*. (B) In the standard MR framework where marginal genetic effects are modeled, horizontal pleiotropy $\alpha_{j}$ consists of both LD-induced (dashed lines between $Z_{j}$ and $G_{j}$’s represent their correlation) and biological pleiotropy (arrow from $Z_{j}$ to *Y*). In the proposed CMR framework, LD-induced pleiotropy is adjusted for in stage 1 through the modeling of conditional genetic effects ($\beta_{Xj}^{*}$ and $\beta_{Yj}^{*}$). Then biological pleiotropy $\alpha_{j}^{*}$ is accounted for in stage 2 through the use of robust MR methods.

Mendelian randomization (MR) is increasingly used in epidemiological studies to assess the causality of an exposure for an outcome using observational data. It overcomes the challenge of the no-confounding assumption in traditional observational studies by using genetic variants as instrumental variables (IVs). The development of summary-level MR, which requires only genome-wide association study (GWAS) summary statistics further expands its applicability. The validity of MR analysis relies on three IV assumptions: (1) the genetic instrument is associated with the exposure; (2) the genetic instrument is not associated with confounders of the exposure-outcome relationship; and (3) the genetic instrument is only associated with the outcome through the exposure. In practice, however, the validity of the third assumption is often challenged due to widespread horizontal pleiotropy—genetic variants influencing the outcome through pathways other than the exposure. Horizontal pleiotropy can manifest in various forms in MR.^1^ For instance, the genetic instrument may influence the outcome directly or through a confounder. In addition, it may be confounded by linkage disequilibrium (LD); that is, a nearby single nucleotide polymorphism (SNP) is associated with the outcome, and due to the correlation with this SNP, the instrument is not independent of the outcome conditional on the exposure (Figure 1A).

One type of approach to address pleiotropy in MR is to simply remove potential pleiotropic (invalid) IVs. For example, one may search for potential pleiotropic pathways using PhenoScanner,^2^ and remove IVs associated with plausible confounders from the analysis. Steiger filtering can also be used to identify horizontally pleiotropic variants to be removed from the analysis if their association with the outcome is stronger than that with the exposure.^3^ Another type of approach is to adjust for pleiotropic pathways such that pleiotropic IVs may become valid. For example, when the IV is associated with other phenotypes that influence the outcome, including these phenotypes as additional exposures in multivariable MR (MVMR)^4^ can adjust for horizontal pleiotropy. However, some of these approaches require a good understanding of the biological mechanism underlying the exposure-outcome relationship to accurately identify confounders or pleiotropic pathways.

In addition to the above approaches to directly remove pleiotropic IVs or adjust for pleiotropic pathways, many robust MR methods have been proposed without any understanding of biological mechanism. These methods can provide valid inference even when the “no horizontal pleiotropy” assumption is violated, albeit relying on other untestable assumptions. Some methods make assumptions on the distribution of horizontal pleiotropy, such as the Instrument Strength Independent of Direct Effect (InSIDE) assumption and/or the balanced pleiotropy assumption (i.e., the average of horizontal pleiotropic effect is zero), etc. Notable methods include inverse-variance weighting (IVW),^5^ Egger regression,^6^ robust adjusted profile score method (RAPS),^7^ among others. Other methods require some IVs to be valid, relying on the majority valid assumption (e.g., weighted-median^8^) or plurality valid assumption (e.g., weighted-mode,^9^ cML^10^). For a more detailed review, we refer readers to Burgess et al.^11^ and Sanderson et al.^1^ Nevertheless, the pervasiveness of horizontal pleiotropy and its manifestation in diverse forms continue to render these untestable assumptions vulnerable, posing a challenging task for these robust MR methods.

Here, we introduce a two-stage framework called CMR which combines conditional analysis and **MR**, to automatically adjust for pleiotropic pathways in MR analysis without the need for biological understanding. In stage 1, we propose a conditional genetic effect model incorporating genetic variants proximal to the IVs such that pleiotropy induced by LD will be removed. The remaining horizontal pleiotropy is then accounted for in stage 2 by the use of robust MR methods. We would like to emphasize that CMR is a universal polygenic MR framework that accommodates the application of many existing robust summary-level polygenic MR methods, and as we show later, it improves their robustness to horizontal pleiotropy. Moreover, it only utilizes GWAS summary statistics and an LD reference panel. In this paper, we conduct extensive simulation studies to show that the performance of various robust MR methods is greatly improved when implemented within the proposed CMR framework. We then apply the proposed framework to a negative control outcome analysis, demonstrating its better type-I error control in practice. We further explore the performance of CMR in identifying potential causal relationships through the application of body mass index on multiple diseases. We also provide a discussion on the comparison of CMR and other related methods.

## Results

### Overview of the proposed CMR framework

While vertical pleiotropy (Figure 1A) is the very phenomenon upon which MR relies, horizontal pleiotropy has been a major concern in MR due to its potential to bias the causal inference. Horizontal pleiotropy is the phenomenon that a genetic variant affects the outcome independently of the exposure of interest, which can manifest in different forms. The classic horizontal pleiotropy, also referred to as **biological** (horizontal) pleiotropy (Figure 1A) throughout, is where the genetic instrument is independently associated with the outcome either directly or indirectly through other trait(s). Another perhaps more prevalent source of horizontal pleiotropy in MR occurs when another SNP (*G*), in LD with the IV (*Z*), is associated with the outcome.^12^^,^^13^^,^^14^ Due to the correlation between *Z* and *G*, the IV is also associated with the outcome independently of the exposure, and we refer it to as **LD-induced** (horizontal) pleiotropy (Figure 1A). These two types of horizontal pleiotropy can happen together as well.

Motivated by the presence of different forms of horizontal pleiotropy, we propose a simple and universal framework called CMR to address them in two stages. Different from the standard MR framework that models the GWAS marginal genetic effects, the proposed CMR framework models the conditional genetic effects in MR (Figure 1B; material and methods). In the first stage, LD-induced pleiotropy is adjusted by a conditional analysis framework. Briefly, for each IV $Z_{j}$ used in the standard MR framework, in step 1, we search for neighboring SNPs that *jointly affect the outcome*. Then in step 2, we adjust for this LD-induced pleiotropy by calculating the *conditional* genetic effect of the $Z_{j}$ on *X* and *Y* conditioning on these neighboring SNPs. These two steps are implemented using the COJO method.^15^ In the second stage, robust MR methods are applied using the conditional genetic effect estimates from stage 1. Given the additional stage 1 to guard against LD-induced pleiotropy compared with the standard MR framework, when applying MR methods in stage 2, a valid IV can now be viewed as one without *biological* horizontal pleiotropy rather than excluding all forms of horizontal pleiotropy. This often results in an increased number of valid IVs to be used in the MR methods, thus improving their performance.

In this paper, the prefix “MR-“ will indicate methods implemented in the standard MR framework using GWAS marginal genetic effect estimates, while “CMR-” will indicate methods implemented in the CMR framework using conditional genetic effect estimates.

### Simulations: Improved performance of various MR methods using the proposed CMR framework

**Figure 2:**
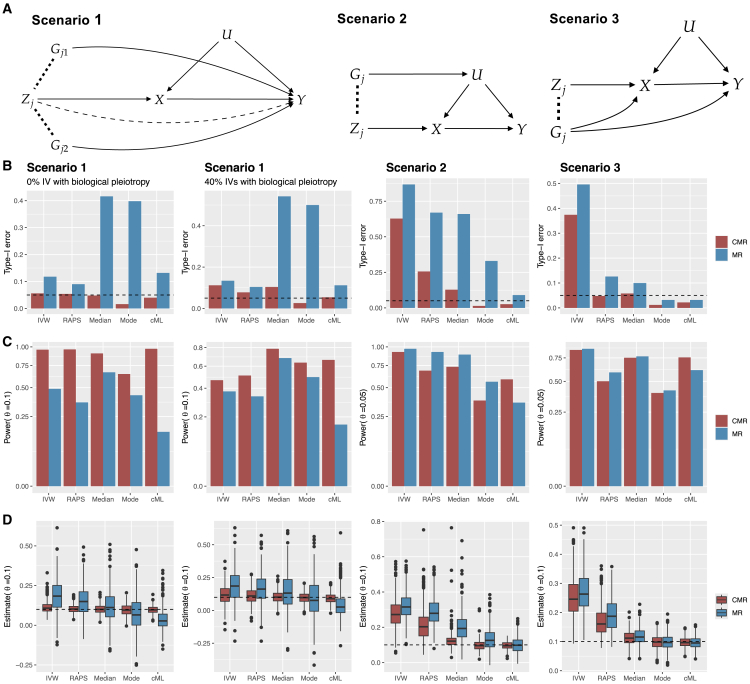
Simulations across different scenarios (A) Directed acyclic graphs illustrating three data-generating scenarios (see simulation setups for full details). Dashed lines represent the correlation between SNPs. In scenario 1, the proportion of IV with biological pleiotropy varied from 0% to 60% (dashed arrow from $Z_{j}$ to *Y*). (B–D) The first two columns correspond to two cases in scenario 1, with 0% and 40% $Z_{j}$’s having biological pleiotropy respectively. The third and fourth columns correspond to scenarios 2 and 3. From top to bottom are type-I errors (when θ=0), power and boxplots of estimates across 500 replicates.

We simulated 20 LD-independent candidate IVs and considered three different scenarios that horizontal pleiotropy could occur (Figure 2A; and see simulation setups for data generation details). For each simulated dataset, we performed LD clumping to select IVs and assessed the performance of five MR methods—IVW, RAPs, weighted-median, weighted-mode, and cML—implemented within both the standard MR and the proposed CMR frameworks. Figure 2B, from top to bottom, shows empirical type-I errors, power, and boxplots of point estimates across various scenarios. Detailed results are available in Tables S1–S6.

In the first scenario, when all 20 IVs had two neighboring variants that directly affected the outcome with positive direct effects (first column in Figure 2A), the balanced pleiotropy assumption required by MR-IVW and MR-RAPS was likely to be violated; and since all IVs were theoretically invalid due to LD-induced pleiotropy, the majority assumption required by MR-median and the plurality assumption required by MR-mode and MR-cML were also violated. We furthermore varied the proportion of IVs exhibiting biological pleiotropy, from 0%, 20%, 40%, and 60% (dashed arrow in Figure 2A scenario 1). As shown in the first two columns in Figure 2B (corresponding to 0% and 40% biological pleiotropy respectively), all five MR methods had inflated type-I errors and biased estimates when implemented in the standard MR framework, while all methods displayed better-controlled type-I errors and less biased estimates in CMR when the LD-induced pleiotropy was adjusted. In particular, in the case of 0% IV with biological pleiotropy (Figure 2B column 1), CMR methods performed well with almost unbiased estimates, well-controlled type-I error and higher power, which was expected as all IVs would become valid in CMR stage 2 after the adjustment for LD-induced pleiotropy. As the number of IVs with biological pleiotropy increased, some CMR methods started to have inflated type-I errors, with CMR-cML performing the best overall.

In scenario 2, *all* 20 candidate IVs $Z_{j}$’s had a neighboring variant affecting the outcome through the hidden confounder, thus all of them had LD-induced pleiotropy. In scenario 3, *half* of the $Z_{j}$’s had LD-induced pleiotropy with a neighboring variant $G_{j}$ affecting both *X* and *Y*, while the remaining half were valid without any types of horizontal pleiotropy. All MR methods exhibited inflated type-I errors and biased estimates (Figure 2B columns 3, 4), except for MR-mode and MR-cML in scenario 3. This was expected given that only the plurality assumption was met in the standard MR framework in scenario 3. Notably, following LD-induced pleiotropy adjustment in CMR, the performance of all methods exhibited varying degrees of improvement (with less inflated type-I errors and smaller bias). It might seem surprising that, CMR-IVW, CMR-RAPS and CMR-Median still had unsatisfactory performance. This was because a neighboring variant $G_{j}$ affecting the confounder (and thus affecting the exposure in scenario 2) or affecting the exposure (in scenario 3) may be selected as the IV, which introduced biological pleiotropy that these three methods failed to account for. For example, in scenario 2, when such $G_{j}$’s were selected as IVs, the InSIDE assumption required by IVW and RAPS was violated in the second stage of CMR. However, when LD-induced pleiotropy was removed, which made some IVs (those selected $Z_{j}$’s) free of pleiotropy and the plurality assumption still hold in stage 2, CMR-Mode and CMR-cML performed well in scenario 2. As shown in Figure S1, when we applied CMR only using the 20 $Z_{j}$’s as IVs, all CMR methods had well-controlled type-I errors and unbiased estimates.

In scenarios 1 and 2, we also varied the proportion of IVs with LD-induced pleiotropy from {0%,20%,40%,60%,80%}. Detailed results are provided in Tables S7–S15. When no invalid IVs had LD-induced pleiotropy, MR and CMR methods gave the same results. This was expected, as no outcome-associated SNPs were selected in stage 1 in CMR; therefore, the marginal genetic effect estimates were the same as the conditional genetic effect estimates. On the other hand, in the presence of invalid IVs with LD-induced pleiotropy, CMR always improved performance over the standard MR framework in the considered experiments, with better-controlled type-I error, higher power, and smaller bias and MSE. Additionally, we reduced the SNP-exposure effect sizes such that the F-statistics were similar to those in the real data applications (see scenario 4 in simulation setups). As shown in Tables S16 and S17, we reached a similar conclusion that after adjusting for LD-induced pleiotropy, CMR had more improved performance than the standard MR framework.

Overall, in all scenarios considered, all five MR methods examined here had better performance in the proposed CMR framework than the standard MR implementation, as demonstrated by better type-I error control, smaller bias and mean squared error, and oftentimes higher power. Additionally, it is worth noting that the conditional analysis in stage 1 was designed to adjust for LD-induced horizontal pleiotropy. If IVs used in the analysis exhibited biological horizontal pleiotropy, then CMR relied on the use of robust MR methods to further account for that, where CMR-cML outperformed the other four methods with well-controlled type-I error and the smallest MSE in a wide range of scenarios.

### Real data application: Negative control outcome analysis

In the first application, we conducted a negative control outcome analysis to evaluate if CMR can better control type-I error, where the outcomes were determined before the exposure and were not expected to be affected by any of the exposures considered.^16^ We considered a wide range of exposures, including seven diseases of the circulatory system, three diseases of the digestive system, three mental and behavioral disorders, Alzheimer disease, type 2 diabetes (T2D), and body mass index (BMI). On the outcome side, we chose natural hair color before graying (hair color: black, hair color: blonde, hair color: light brown, hair color: dark brown), and birth weight as they are largely determined at birth. We also considered standing height, which is primarily determined by genetics, nutrition, and hormonal factors during growth phases, while the exposures considered were mostly developed in the latter course of life. In total 16×6=96 MR analyses were performed, and we applied IVW, RAPS, weighted-median, weighted-mode, and cML in both the standard MR framework with GWAS marginal estimates as input and CMR framework with COJO conditional estimates as input. The number of IVs ranged from 6 to 350, with a median of 53; and the F-statistics^17^ in the standard MR framework ranged from 37 to 112, with a median of 55 (numbers were rounded to integer). The number of IVs being adjusted for LD-induced pleiotropy in CMR ranged from 0 to 269 (the proportion ranged from 0% to 100%, with a median of 22%; see Figure S5). After adjusting for LD-induced pleiotropy, the F-statistics ranged from 33 to 118, with a median of 53.

**Figure 3:**
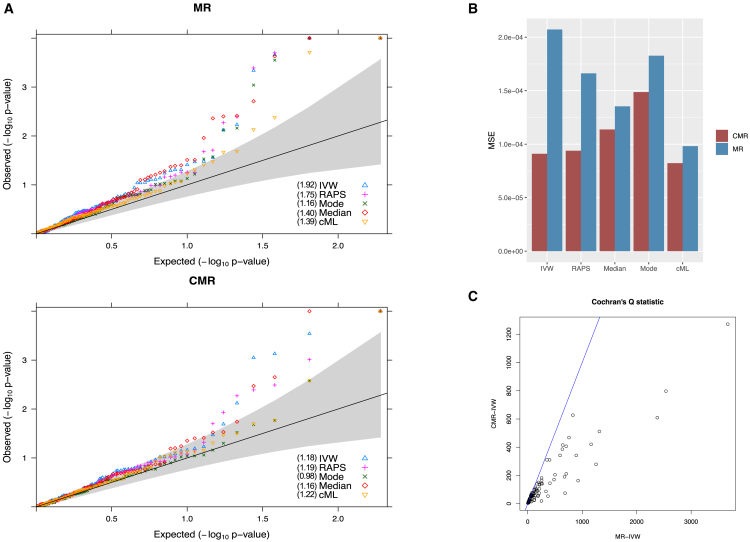
Negative control outcome analysis (A) Q-Q plots of $-\log_{10}\left(p\right)-\text{values}$ from five methods using standard MR (top) and CMR (bottom) frameworks. p−values are truncated at $1\times 10^{-4}$. Numbers in parentheses are inflation factors. (B) Mean squared error of 96 causal estimates assuming the true effect size is zero. (C) Cochran’s Q statistics computed in MR-IVW (X axis) versus CMR-IVW (Y axis). Blue line is the 45° diagonal line.

Figure 3A shows the Q-Q plots of the five MR methods using the standard MR (top) framework and CMR framework (bottom), respectively. First and foremost, consistent with our simulation studies, it is evident that all methods controlled the type-I error better in the CMR framework, with weighted-mode and cML performing the best with the least inflation. A direct comparison between each method within both the standard MR and CMR frameworks is provided in Figure S2. Figure 3B shows the MSE for the 96 causal effect estimates under the assumption of null effect. All methods displayed a smaller MSE using the proposed CMR framework. We also found that the estimated standard errors of the causal parameter were smaller (i.e., more precise) in CMR methods than their standard MR-counterparts. Next, we compared Cochran’s Q statistics for the MR-IVW and CMR-IVW models. Cochran’s Q statistic can be used for detecting heterogeneity among the ratio causal estimates, where excessive heterogeneity indicates the possible presence of horizontal pleiotropy.^18^ As shown in Figure 3C, after adjusting for LD-induced pleiotropy, CMR dramatically reduced the heterogeneity presented in the data. Furthermore, we used Steiger filtering to exclude potential invalid IVs and then applied the five standard MR methods. The results were comparable to those obtained without Steiger filtering in Figure 3A top panel, which exhibited more inflation than the proposed CMR. We relegated the detailed results to Figure S2. We also implemented CMR with different *p* value thresholds to select outcome-associated SNPs in the stage 1 conditional analysis. As shown in Figure S3, using a less stringent threshold (e.g., 5e−4) generally resulted in less inflation of CMR methods in the negative control analysis, potentially because more IVs were adjusted for LD-induced pleiotropy; using a more stringent threshold (e.g., 5e−8) made CMR perform more similarly to the standard MR.

### Real data application: Causal role of BMI in multiple diseases

Obesity is a global epidemic that could increase the risk of a wide range of diseases. In this application, we used BMI as a proxy marker for obesity to investigate its causal role in 14 diseases using the standard MR and the proposed CMR framework. The number of LD-independent IVs for BMI used in the standard MR analysis is 453, with a median F-statistics of 52. Due to possible collinearity in COJO conditional analysis, the number of IVs used in the CMR analysis ranged from 441 to 453. And the F-statistics ranged from 45 to 52, with a median of 52.

**Figure 4:**
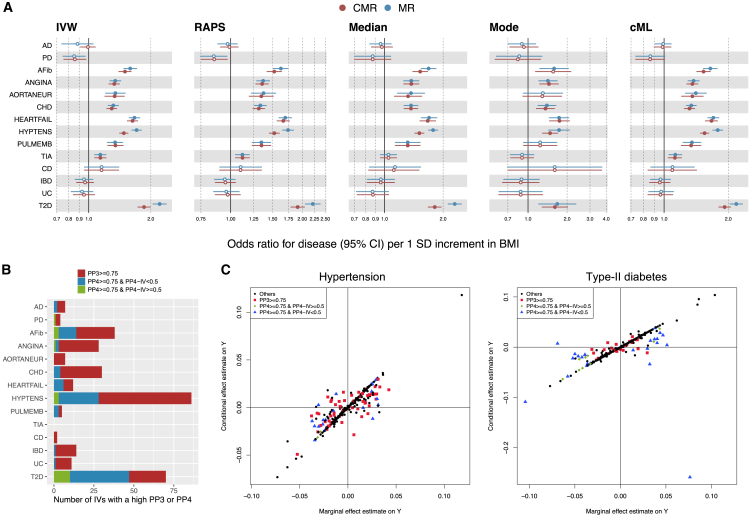
Investigation of the role of BMI in multiple diseases (A) Forest plots of causal effect estimates (x axis) of BMI on multiple diseases (y axis) obtained from different methods in both the standard MR and CMR frameworks. Solid circles represent statistically significant results after Bonferroni correction with p≤0.05/14. (B) Number of IVs with evidence against or for colocalization based on COLOC results. (C) Comparison of marginal genetic effect estimates (x axis) and conditional genetic effect estimates (y axis) on *Y*. Left: Hypertension. Right: Type 2 diabetes. AD, Alzheimer disease; PD, Parkinson disease; AFib, atrial fibrillation; ANGINA, angina pectoris; AORTANEUR, aortic aneurysm; CHD, coronary heart disease; HEARTFAIL, heart failure; HYPTENS, hypertension; PULMEMB, pulmonary embolism; TIA, transient ischemic attack; CD, Crohn disease; IBD, inflammatory bowel disease; UC, ulcerative colitis; T2D, type 2 diabetes.

First, consistent across five standard MR and CMR methods, BMI was not causally associated with the two nervous system diseases (Alzheimer disease and Parkinson disease), and the three digestive system diseases (Crohn disease, inflammatory bowel disease and ulcerative colitis) after Bonferroni correction with p≤0.05/14. On the other hand, studies have shown that obesity leads to the development of cardiovascular disease and directly contributes to various cardiovascular risk factors, including T2D and hypertension.^19^ Our analysis confirmed statistically significant causal effects of BMI on all eight examined circulatory system diseases and T2D as indicated by IVW, RAPS, and cML in both the CMR and standard MR frameworks (Figure 4A). Weighted-Median also detected these effects in the same direction, but the estimated effect size of BMI on transient ischemic attack was smaller and not significant. Weighted-Mode identified fewer diseases that BMI might causally affect, possibly due to its lower power in detecting causal effects compared to other MR methods, as demonstrated in previous simulation studies and literature.^9^

Second, we noted that the causal effect estimates of BMI on hypertension and T2D were notably different between the CMR and the standard MR methods. This suggested a potentially significant impact of LD-induced horizontal pleiotropy in the analyses of BMI-hypertension and BMI-T2D. To partially validate this hypothesis, we performed colocalization analyses for the 14 BMI-disease pairs. Colocalization is designed to explore if a pair of traits share causal SNPs, and it can be used as a sensitivity analysis to evaluate MR assumptions.^20^ Specifically, each of the IVs and its neighboring markers (distance within 200 kb) were used to define a test region, and we applied the Bayesian colocalization method COLOC^21^ on each region (refer to colocalization analysis using COLOC for details). Our primary interest was the posterior probability (PP)_3_ of distinct causal variants for BMI and the disease and the PP_4_ of a shared causal variant affecting both traits (i.e., colocalized). Furthermore, for colocalized regions (with PP_4_ ≥0.75), we calculated the PP_4_-IV of the IV being causal to both traits. A high PP_3_ may indicate that the IV was in LD with the other potentially causal variant of the disease, and thus introducing the LD-induced pleiotropy (Figure 1A column 3). On the other hand, a high PP_4_ suggested colocalization within a region; however, if it were not colocalized at the IV used in the MR analysis (PP_4_-IV <0.5), but rather at a neighboring variant, it could also introduce the LD-induced pleiotropy as we’ve seen in simulation scenario 3 (Figure 2A column 3). As confirmed in Figure 4B, pairs BMI-hypertension and BMI-T2D had the highest number of IVs with either a high PP_3_ (at the corresponding region), or a high PP_4_ but a low PP_4_-IV, while other pairs had less than 10% of such IVs. Adjusting for these LD-induced pleiotropy effects (see Figure S6), four methods (except for weighted-mode) implemented in CMR gave considerably diminished estimates for the causal effects of BMI on hypertension and T2D compared with standard MR (Figure 4A).

To further gain an exploratory sense of the reason for the difference between CMR and MR results for BMI-hypertension and BMI-T2D, we plotted the marginal against the conditional genetic effect estimates of the IVs on the outcome in Figure 4C. The exact numbers of IVs being adjusted for LD-induced pleiotropy for each pair are provided in Figure S6. While the majority of the IVs had similar marginal and conditional genetic effect estimates, CMR effectively adjusted for LD-induced pleiotropy as highlighted by red squares (IVs with high PP_3_) and blue triangles (IVs with high PP_4_ but low PP_4_-IV). Consequently, heterogeneity in the data was substantially reduced as suggested by Cochran’s Q statistics decreasing from 1418 in standard MR to 971 in CMR for BMI-hypertension, and from 1384 to 975 for BMI-T2D. We would like to highlight a few points here. First, instead of simply removing potentially invalid IVs, CMR tried to adjust for LD-induced horizontal pleiotropy such that useful information would remain in the conditional genetic effect estimates. As a sensitivity analysis, we additionally performed the standard MR analysis after removing potentially invalid IVs using Steiger filtering. We found that causal effect estimates of BMI on both traits were also attenuated compared with the standard MR analysis with the full set of IVs, but with varying degrees of attenuation compared with CMR (Figure S3). Second, IVs with a high PP_4_-IV may indicate biological horizontal pleiotropy or vertical pleiotropy (green diamonds), for which stage 1 of CMR was not designed to adjust. However, some were still being adjusted in the analysis. This could be attributed to our use of larger genomic regions (based on independent LD blocks) in CMR than those used in the COLOC analysis, where pleiotropy may still be introduced via LD with variants beyond 200 kb. For the same reason, some IVs without evidence for or against colocalization (black dots) were also adjusted for LD-induced pleiotropy by CMR (see Figure S6). Another possible reason is that, COLOC typically estimates a high PP_3_ or PP_4_ only if there are variants strongly (marginally) associated with both the exposure and the outcome, while joint analysis performed in step 1 in CMR is sometimes more powerful than the single-variant (marginal) analysis.^15^

## Discussion

Despite the development of robust MR methods, most of them are focused on relaxing the “no horizontal pleiotropy” assumption by replacing it with other untestable assumptions, either on the distribution of pleiotropic effect, or on the number/proportion of invalid IVs. Originally proposed to model the marginal GWAS effects, these MR methods need to address a mix of various types of horizontal pleiotropy including both biological pleiotropy and LD-induced pleiotropy. Consequently, they become vulnerable to the violation of, for instance, InSIDE or majority assumptions. In this work, we have proposed the two-stage CMR framework where LD-induced pleiotropy is removed in the first stage, and in the second stage, robust MR methods are applied to account for the remaining pleiotropy. Through extensive simulation studies and the two real data applications, we have shown that the proposed CMR framework significantly improved several existing MR methods.

Our work is motivated by a well-known fact in MR that horizontal pleiotropy can arise due to the correlation between IVs and other SNPs that independently influence the outcome.^22^ This phenomenon is not uncommon. For example, in a study analyzing four autoimmune diseases, Fortune et al.^12^ found that nearly half of the overlapping and genome-wide significant loci correspond to distinct causal variants. Pickrell et al.^13^ also noted a considerable presence of nearby signals influencing two traits in an analysis of 42 traits. Moreover, van Der Graaf et al.^14^ demonstrated that expression quantitative trait loci (eQTLs) linked to a specific gene are very likely to exhibit LD with, but not necessarily overlap with, eQTLs of other genes. In our data applications, we’ve also observed a similar phenomenon, and CMR adeptly addressed these potential LD-induced pleiotropic effects in the conditional analysis stage.

A crucial feature of CMR is that, instead of simply removing horizontally pleiotropic variants, it attempts to adjust for these effects. This enables IVs with only LD-induced horizontal pleiotropy to be treated as valid in the subsequent MR analysis. Furthermore, it avoids several limitations of current pleiotropic variant exclusion practices. For example, Steiger filtering is designed to remove IVs with horizontal pleiotropy due to reverse causality, while IVs with other forms of pleiotropy may remain. As shown in our negative control outcome application, results with and without Steiger filtering had similar inflation. Cherry-picking of IV based on other criteria, such as its significant association with other traits, or its contribution to heterogeneity may generate bias or over-precision in the causal estimate.^23^

MVMR^4^ and MR-TRYX^23^ are two other approaches that also adjust for pleiotropic pathways instead of removing IVs. Rather than conditioning on proximal genetic variants as in CMR, MVMR and MR-TRYX attempt to adjust for horizontal pleiotropic pathways through conditioning on related exposures. Despite the potential to correct for certain biological pleiotropy, both approaches rely on these pleiotropic pathways to be accurately identified and incorporated in the analysis, which, however, is unrealistic in practice. In contrast, CMR does not require such a biological understanding of the variant-exposure-outcome pathways. It is an automated process only relying on the LD information among variants. Furthermore, CMR accommodates the use of robust MR methods in stage 2 to further account for remaining (biological) pleiotropy.

There are also differences between the proposed CMR and another method, MR-Link,^14^ which shares the same idea as CMR to correct for LD-induced pleiotropy. First, MR-Link is proposed for the investigation of a molecular trait (e.g., gene expression) on an outcome. It focuses on a specific genomic region and uses correlated *cis*-SNPs as IVs; while CMR is developed in a polygenic MR context that uses SNPs in multiple regions across the whole genome. Second, CMR uses COJO to identify variants jointly associated with the outcome while MR-Link first includes all proximal SNPs and then performs LD-pruning and fits a ridge regression with IVs and all LD-pruned SNPs. Of particular significance, MR-Link requires the individual-level outcome GWAS data to adjust for LD-induced pleiotropy, whereas CMR only uses summary statistics and an LD reference panel, making our proposed framework more readily applicable. Finally, MR-Link assumes that horizontal pleiotropy is explained by variants in LD with the IVs, and thus not allowing the presence of biological pleiotropy. Instead, CMR is a universal framework wherein most of the established robust MR methods can be applied, thereby further enhancing its robustness to invalid IVs with biological pleiotropy.

Several limitations of the proposed framework are worth noting. First, CMR is currently built upon the two-sample MR setup with independent SNPs as IVs, which is reflected in the use of various two-sample MR methods in the second stage. Theoretically, MR methods that allow for sample overlap between two GWAS datasets^24^^,^^25^ can also be implemented in the second stage. These MR methods usually require the input of a correlation matrix capturing the correlation between the two sets of GWAS marginal estimates, which can be estimated by the bivariate LD score regression. However, in CMR where a conditional genetic effect model is used, such a correlation matrix needs to be adjusted accordingly. Another future direction is to incorporate MR methods that permit the use of correlated IVs.^26^^,^^27^ This is of particular importance in *cis*-MR study where genetic variants are all in the same gene region and LD-induced pleiotropy may be more prevalent. Second, while the conditional analysis framework can reduce LD-induced horizontal pleiotropy, there is also a risk of weakening some IVs’ associations with the exposure. In two-sample MR, the weak IV bias is expected to be toward the null, but not necessarily in the case with overlapping samples.^28^ Applying methods less sensitive to weak IVs, such as cML and RAPS, which do not rely on the assumption of no measurement error in the SNP-exposure association (NOME), can mitigate this issue. In our applications, weak IV is less of a concern as indicated by large F-statistics and that most IVs remained genome-wide significant even after adjusting for nearby variants in CMR. Nevertheless, caution should be taken if weak IVs substantially impact the analysis. Finally, more applications of CMR on real data and MR methods are warranted.

In conclusion, we have introduced CMR as a two-stage framework to tackle the pervasive horizontal pleiotropy in MR, which aims to separate LD-induced and biological pleiotropy into two stages. Our strategy is novel compared with the standard MR framework in its model of conditional genetic effects of IVs on exposure and outcome adjusting for nearby variants, and thus improving the robustness of various existing MR methods to invalid IVs in practice. We expect our framework can serve as an important sensitivity analysis tool for MR analysis.

## Material and methods

### A standard two-sample MR framework with independent genetic instruments

In a standard MR framework with *m* independent SNPs, $Z_{1},\ldots ,Z_{m}$, as IVs, the relationship between the *j*-th genetic instrument, the exposure *X*, the outcome *Y* and the correlated errors $e_{X}$ and $e_{Y}$ is depicted in Figure 1B top left panel. Without loss of generalization, assuming *X*, *Y*, $Z_{j}$ are all standardized to have mean zero and variance one, the structural equation model is given as follows:

(Equation 1) $$X=\beta_{Xj}Z_{j}+e_{X}\text{,}$$

$$Y=\theta X+\alpha_{j}Z_{j}+e_{Y}=\underset{\beta_{Yj}}{\underset{︸}{\left(\theta \beta_{Xj}+\alpha_{j}\right)}}Z_{j}+\left(\theta e_{X}+e_{Y}\right)\text{,}$$

where θ is the causal effect of interest, $\alpha_{j}$ is the horizontal pleiotropy effect, and $\beta_{Xj}$ and $\beta_{Yj}$ are the total/marginal effects of $Z_{j}$ on *X* and *Y*, respectively. The random errors $e_{X}$ and $e_{Y}$ are correlated due to the presence of unmeasured confounding. From Equation 1, we have the key relationship used in most of the summary-level MR methods:

(Equation 2) $$\beta_{Yj}=\theta \beta_{Xj}+\alpha_{j}\text{.}$$

Throughout the paper, we consider the two-sample MR setup, where the GWAS summary-level estimates (and corresponding standard errors) for $\beta_{Xj}$ and $\beta_{Yj}$ are obtained from two independent/non-overlapping samples, denoted as ${\left\{{\hat{\beta }}_{Xj},{\hat{\sigma }}_{Xj},{\hat{\beta }}_{Yj},{\hat{\sigma }}_{Yj}\right\}}_{j=1}^{m}$. When $\alpha_{j}=0$ and $\beta_{Xj}\neq 0$, $\theta =\beta_{Yj}/\beta_{Xj}$ can be consistently estimated by the Wald-ratio estimator ${\hat{\beta }}_{Yj}/{\hat{\beta }}_{Xj}$. In the presence of horizontal pleiotropy effect, different robust MR methods may handle it differently, for example, by putting distribution assumption on $\alpha_{j}$, or trying to identify and remove invalid IVs with a non-zero $\alpha_{j}$.

### Motivation: Horizontal pleiotropy induced by LD

While it is plausible that the genetic instrument associated with the exposure is also causally associated with the outcome (not through the exposure), another likely scenario of the presence of horizontal pleiotropy is due to the LD between the genetic instrument and the neighboring variants that are causally associated with the outcome. For simplicity, suppose that in Figure 1B bottom left panel, one $G_{j}$ that is in LD with $Z_{j}$ has a direct effect on the outcome *Y*, and as a result $Z_{j}$ will have a direct effect on *Y* if modeled marginally in Figure 1B top left panel. To be specific, we may write the model as follows:

$$X=\beta_{Xj}Z_{j}+\epsilon_{X}\text{,}$$

$$Y=\theta X+r_{j}G_{j}+\epsilon_{Y}\text{.}$$

In GWAS summary statistics, the marginal genetic effects of $Z_{j}$ are estimated by ${\hat{\beta }}_{Xj}={\left(Z_{j}^{\prime }Z_{j}\right)}^{-1}\left(Z_{j}^{\prime }X\right)$ and ${\hat{\beta }}_{Yj}={\left(Z_{j}^{\prime }Z_{j}\right)}^{-1}\left(Z_{j}^{\prime }Y\right)$, and thus after simple algebra we have $E\left[{\hat{\beta }}_{Xj}\right]=\beta_{Xj}$ and $E\left[{\hat{\beta }}_{Yj}\right]=\theta \beta_{Xj}+r_{j}{\left(Z_{j}^{\prime }Z_{j}\right)}^{-1}\left(Z_{j}^{\prime }G_{j}\right)$. Assuming genotypes $Z_{j}$ and $G_{j}$ are both standardized, then $E\left[{\hat{\beta }}_{Yj}\right]=\theta \beta_{Xj}+r_{j}\rho_{j}$, where $\rho_{j}$ is the LD correlation between SNPs $Z_{j}$ and $G_{j}$. As a result, in the standard MR framework discussed before, we have horizontal pleiotropy $\alpha_{j}=r_{j}\rho_{j}$ in Equation 2.

If furthermore $G_{j}$ has an effect on *X* or the confounding *U*, correlated pleiotropy is induced and the InSIDE assumption will be violated as shown in Figure 2A scenarios 2 and 3. However, one straightforward but important observation is that, in both cases, $Z_{j}$ does not have a direct pathway to *Y* conditional on $G_{j}$. That is, if we let $\beta_{Xj}^{*}$ and $\beta_{Yj}^{*}$ be the conditional effect of $Z_{j}$ on *X* and *Y* respectively conditional on $G_{j}$, then $\beta_{Yj}^{*}=\theta \beta_{Xj}^{*}$ and $Z_{j}$ can be treated as a valid IV again. We generalize this idea and propose a new framework for MR next.

### CMR: A new framework by modeling conditional genetic effect in MR

Motivated by the simple example above, we consider a more general model between the *j*-th genetic instrument $Z_{j}$, the exposure *X*, the outcome *Y*, and neighboring genetic variants $G_{j}$ affecting *Y* (either directly, through *X*, or through unmeasured confounding), and correlated errors $\epsilon_{X}$ and $\epsilon_{Y}$ in Figure 1B bottom left panel. Without loss of generalization, assuming *X*, *Y*, $Z_{j}$, and $G_{j}$ are all standardized to have mean zero and variance one, we have

(Equation 3) $$X=\beta_{Xj}^{*}Z_{j}+G_{j}\gamma_{j}+\epsilon_{X}\text{,}$$

$$Y=\theta X+\alpha_{j}^{*}Z_{j}+G_{j}r_{j}+\epsilon_{Y}$$

$$=\underset{\beta_{Yj}^{*}}{\underset{︸}{\left(\theta \beta_{Xj}^{*}+\alpha_{j}^{*}\right)}}Z_{j}+G_{j}\left(\theta \gamma_{j}+r_{j}\right)+\left(\theta \epsilon_{X}+\epsilon_{Y}\right)\text{.}$$

We have a key relationship used in **CMR**, which is similar to that in Equation 2, but in a conditional effect scale instead of the marginal effect scale:

(Equation 4) $$\beta_{Yj}^{*}=\theta \beta_{Xj}^{*}+\alpha_{j}^{*}\text{,}$$

where the conditional genetic effects $\beta_{Xj}^{*}$ and $\beta_{Yj}^{*}$ can be estimated using GWAS marginal estimates and LD matrix among $Z_{j}$ and $G_{j}$ in COJO.^15^

To see how using Equation 4 can reduce LD-induced horizontal pleiotropy compared with Equation 2, notice that the marginal genetic effects in the marginal model Equation 1 are $\beta_{Xj}=\beta_{Xj}^{*}+\rho_{j}^{\prime }\gamma_{j}$, $\beta_{Yj}=\beta_{Yj}^{*}+\rho_{j}^{\prime }\left(\theta \gamma_{j}+r_{j}\right)=\theta \beta_{Xj}+\left(\alpha_{j}^{*}+\rho_{j}^{\prime }r_{j}\right)$, where $\rho_{j}$ is the correlation between $Z_{j}$ and $G_{j}$, and thus we have $\alpha_{j}=\alpha_{j}^{*}+\rho_{j}^{\prime }r_{j}$. In the absence of biological horizontal pleiotropy ($\alpha_{j}^{*}=0$), horizontal pleiotropy $\alpha_{j}$ in the standard MR framework can still be induced by LD unless $r_{j}=0$ or these effects happen to cancel out with $\rho_{j}^{\prime }r_{j}=0$.

Therefore, with the aim to reduce LD-induced pleiotropy, we propose the CMR framework that models the **conditional** genetic effect of IV conditioning on neighboring genetic variants having an effect on the outcome. It only requires the full GWAS summary statistics for the exposure and the outcome, as well as an LD reference panel. Briefly, we start by selecting independent IVs $Z_{1},\ldots ,Z_{m}$ using LD clumping as in the standard MR framework. Then we search for genetic variants proximal to the IVs that are jointly associated with the outcome and adjust for the GWAS estimates of IVs on *X* and *Y* by conditioning on these extra nearby genetic variants. Finally, we apply existing robust MR methods with these conditional genetic effect estimates to further account for residual horizontal pleiotropy. The procedure is summarized in Algorithm 1.

Algorithm 1 **CMR: A two-stage framework with automated pleiotropy adjustment**

**Require:** Full exposure and outcome GWAS summary data, LD reference panel.

1: Apply LD clumping to obtain independent IVs $Z_{1},\ldots ,Z_{m}$.

2: Group IVs based on 1703 independent LD blocks, denoted as $B_{l}$, l=1,…,1703. Stage 1:

3:**for all** $B_{l}\in \left\{B_{l}:\left|B_{l}\right|>0\right\}$ **do**

4:  Apply COJO on outcome GWAS to select the set of SNPs jointly associated with the outcome, denoted as $O_{l}$.

5:  **if** $\left|O_{l}\right|>0$ **then**

6:  Perform COJO of SNPs in $B_{l}\cup O_{l}$ on the exposure and the outcome respectively to obtain $\left({\hat{\beta }}_{Xj}^{*},{\hat{\sigma }}_{Xj}^{*}\right)$ and $\left({\hat{\beta }}_{Yj}^{*},{\hat{\sigma }}_{Yj}^{*}\right)$, $j\in \left\{j:Z_{j}\in B_{l}\right\}$.

7:  **else**.

8:  $\left({\hat{\beta }}_{Xj}^{*},{\hat{\sigma }}_{Xj}^{*},{\hat{\beta }}_{Yj}^{*},{\hat{\sigma }}_{Yj}^{*}\right)=\left({\hat{\beta }}_{Xj},{\hat{\sigma }}_{Xj},{\hat{\beta }}_{Yj},{\hat{\sigma }}_{Yj}\right)$, $j\in \left\{j:Z_{j}\in B_{l}\right\}$.

9:  **end if**.

10: **end for**.

Stage 2:

11: Apply MR methods (e.g., IVW, RAPS, weighted-Mode, etc.) with the conditional genetic effect estimates $\left\{{\hat{\beta }}_{Xj}^{*},{\hat{\sigma }}_{Xj}^{*},{\hat{\beta }}_{Yj}^{*},{\hat{\sigma }}_{Yj}^{*}\right\}$.

12: **return** MR results.

As shown in Algorithm 1, we propose to use the COJO method^15^ in stage 1 to build a conditional genetic effect framework for stage 2 MR. COJO is a conditional and joint association analysis approach using GWAS summary statistics and an LD reference panel. It performs a stepwise procedure to select SNPs that are jointly associated with the trait based on their conditional P−values, and then estimates the joint effects of all selected SNPs.^15^ We implement COJO in CMR for two different goals. For the first step in stage 1 (Figure 1B top right panel), the goal is to identify SNPs jointly associated with the outcome that may introduce LD-induced pleiotropy in MR due to their correlation with the IVs. We group IVs into 1,703 independent LD blocks $B_{l}$ based on LDetect^29^ and apply COJO on the outcome GWAS dataset per LD block to select such jointly significant ($p-\text{value}<5\times 10^{-6}$) SNPs $O_{l}$. For the second step in stage 1 (Figure 1B bottom right panel), the goal is to estimate the conditional effect of IVs on *X* and *Y* respectively conditioning on SNPs identified in the first step. Thus we apply COJO on the exposure GWAS and the outcome GWAS per LD block respectively including SNPs in $B_{l}\cup O_{l}$ to obtain such effect estimates. In practice, IVs in $B_{l}$ may be highly correlated (e.g., ρ>0.9) with SNPs in $O_{l}$, and COJO will fail to estimate the conditional effects due to collinearity. These IVs will be excluded from the subsequent analysis in CMR.

As previously stated, the proposed CMR framework minimizes horizontal pleiotropy induced by LD by blocking paths between IVs and outcome-associated SNPs in stage 1, and it does not reduce the biological horizontal pleiotropy where $Z_{j}$ directly affects the outcome on its own. Nonetheless, its compatibility with most robust MR methods in stage 2 can further handle this residual pleiotropy that is not fully accounted for by the conditional analysis. The assumptions required by different robust MR methods still apply, however, in the conditional effect scale (Equation 4). For example, the balanced pleiotropy assumption now requires $\alpha_{j}^{*}$ to have mean zero. Furthermore, despite the use of conditional genetic effect in CMR stage 2, the causal parameter of interest θ is the same as that in the standard MR framework (Equations 2 and 4), which does *not* depend on which nearby SNPs $O_{l}$ are included in the conditional analysis. The SNPs selected in the conditional analysis only affect the definition of pleiotropic effect $\alpha_{j}^{*}$ in CMR, i.e., how much LD-induced pleiotropy is removed. We also find that CMR is robust to the linearity assumption of the SNP-exposure effect. Further investigation of CMR regarding the linearity of genetic effects can be found in Section S1.

### Simulation setups

We performed simulation studies to compare the performance of existing MR methods using marginal GWAS estimates in the standard MR framework and conditional genetic effect estimates by COJO in the proposed CMR framework. Five robust MR methods were applied, including random-effect IVW, RAPS, weighted-median, weighted-mode and cML.

We extracted genotype data, selected from 20 randomly chosen LD blocks, of 337,392 unrelated individuals of White ancestry in the UK Biobank. After filtering out rare variants (with minor allele frequency less than 0.05), 4,383 SNPs were retained. The number of SNPs in each LD block ranged from 16 to 551, with a median of 173. Then we generated phenotype data from the following model (Figure 2A):

$$U=\sum_{j=1}^{20}G_{j}\phi_{j}+e_{U}\text{,}$$

$$X=\sum_{j=1}^{20}\beta_{Xj}^{*}Z_{j}+\sum_{j=1}^{20}G_{j}\gamma_{j}+U+e_{X}\text{,}$$

$$Y=\theta X+\sum_{j=1}^{20}\alpha_{j}^{*}Z_{j}+\sum_{j=1}^{20}G_{j}r_{j}+U+e_{Y}\text{,}$$

where $e_{U},e_{X},e_{Y}∼N\left(0,1\right)$ independently. One causal variant (candidate IV) per LD block ($Z_{j}$) was randomly chosen, and the corresponding effect size $\beta_{Xj}^{*}∼\text{Uniform}\left(0.1,0.2\right)$. We considered three scenarios:

(1) In scenario 1 (Figure 2A column 1), two nearby SNPs $G_{j}=\left(G_{j1},G_{j2}\right)$ had direct effects on *Y* in 20×p blocks, with p∈{0,0.2,0.4,0.6,0.8,1}, and $r_{1j},r_{2j}∼\text{Uniform}\left(0.1,0.2\right)$ independently for *j* in the selected blocks; $\phi_{j}=\gamma_{j}=0$ for j=1,…,20. We also varied the proportion of $Z_{j}$’s from {0%, 20%, 40%, 60%} having biological pleiotropy 1$\alpha_{j}^{*}∼\text{Uniform}\left(-0.1,0.\right)$.
(2) In scenario 2 (Figure 2A column 2), one nearby SNP $G_{j}$ had an effect on *U* in 20×p blocks, with p∈{0,0.2,0.4,0.6,0.8,1}, and $\phi_{j}∼\text{Uniform}\left(0.05,0.15\right)$ independently for *j* in the selected blocks; and $\alpha_{j}^{*}=r_{j}=\gamma_{j}=0$ for 0j=1,…,2.
(3) In scenario 3 (Figure 2A column 3), one nearby SNP $G_{j}$ had effects on *X* and *Y* in the first 10 blocks, with $\gamma_{j}∼\text{Uniform}\left(0.05,0.15\right)$ and $r_{j}∼\text{Uniform}\left(0.1,0.2\right)$, j=1,…,10; the rest of IVs were valid with $\gamma_{j}=\alpha_{j}^{*}=r_{j}=0$, j=11,…,20.

The R-squared, defined as $Var\left(\sum \beta_{Xj}^{*}Z_{j}\right)/Var\left(X\right)$, ranged from 0.036 to 0.088, with an average of 0.065. The average F-statistics are over 100 (details are reported in the footnotes of supplementary tables). To mimic the real data applications, we randomly selected 50 LD blocks, and generated one causal variant per LD block with $\beta_{Xj}^{*}∼\text{Uniform}\left(0.02,0.08\right)$ (scenario 4), such that the median F-statistics were around 48 and 45 before and after conditioning respectively. Similar to scenario 1, two nearby SNPs had direct effects on *Y* in 20% or 60% blocks.

In total, 500 replicates were generated in each scenario. To mimic a two-sample setup, we randomly selected two independent sets of 50,000 individuals, and calculated GWAS summary data for the exposure and the outcome respectively using PLINK.^30^ We used all available unrelated individuals with White ancestry as the LD reference panel. LD clumping was applied in each block to select independent IVs with GWAS $p-\text{value}<5\times 10^{-8}$. Then we applied standard MR methods with the selected IVs, as well as the proposed CMR framework.

### Collection of GWAS summary data and preprocessing in real data analysis

In the negative outcome control application, we chose natural hair color before graying (hair color: black, hair color: blonde, hair color: light brown, hair color: dark brown), birth weight, and standing height as our negative outcomes, all of which were retrieved from the Neale Lab round 2 analysis. On the exposure side, we considered a wide range of phenotypes, including eight diseases of the circulatory system (hypertension, atrial fibrillation, coronary heart disease, heart failure, pulmonary embolism, aortic aneurysm, angina pectoris, and transient ischemic attack), two diseases of the nervous system (Alzheimer disease and Parkinson disease), three diseases of the digestive system (Crohn disease, inflammatory bowel disease, and ulcerative colitis), three mental and behavioral disorders (depression, dementia, and alcohol use disorder), T2D, and BMI. To ensure a two-sample design, we retrieved all the exposure GWAS data from FinnGen R9 release.^31^ After the data preprocessing described next, we only found less than three LD-independent IVs for transient ischemic attack and Parkinson disease, which were excluded from the negative control application.

In the second application, we investigated the causal role of BMI in multiple diseases used in the first application, including the eight diseases of the circulatory system, two diseases of the nervous system, three diseases of the digestive system, and T2D. Inverse rank-normal transformed BMI GWAS data were retrieved from the Neale Lab. For all analyses, imputed data from the UK Biobank on unrelated individuals with White ancestry^32^ were used as the LD reference panel.

In the data analysis, we first extracted SNPs common in exposure GWAS, outcome GWAS, and the LD reference panel. Then we selected SNPs genome-wide significantly associated with the exposure ($p-\text{value}<5\times 10^{-8}$) and performed LD clumping ($r^{2}<0.001$, window size 10,000 kb) in PLINK to obtain genetic instruments. Exposure and outcome GWAS data were then harmonized using TwoSampleMR package.^33^ IVW, weighted-median, weighted-mode and cML were implemented in MendelianRandomization package^34^ with default parameters. RAPS was implemented in mr.raps package.

### Colocalization analysis using COLOC

To investigate whether CMR effectively adjusted for LD-induced pleiotropy, we implemented colocalization analysis using COLOC.^21^ COLOC assumes at most one single genetic variant per trait at each test region, and evaluates the PP of every possible configuration: $H_{0}$: no association with either trait; $H_{1}$: association with trait 1 but not trait 2; $H_{2}$: association with trait 2 but not trait 1; $H_{3}$: association with both traits but at different causal variants; $H_{4}$: association with both traits at a shared causal variant. Test regions were defined within 200 kb of the genetic instruments. A high PP_3_ is evidence *against* colocalization, indicating that two different variants causally affect the two traits. As the two variants are usually correlated due to LD, this would typically lead to the violation of no horizontal pleiotropy assumption. A high PP_4_ is evidence *for* colocalization, indicating that a single genetic variant causally affects both traits. We further assessed the posterior probability of the IV being the causal variant, denoted as PP_4_-IV. We used PP_4_-IV ≥0.5 to declare that the IV was the candidate causal variant, which could be vertical pleiotropy, biological pleiotropy, or both. Conversely, a low PP_4_-IV may suggest the presence of horizontal pleiotropy introduced by the LD between the IV and the causal variant. Analysis was implemented using coloc.abf in R package coloc, with default parameters that the prior probability of a variant associated with either of the traits is $1\times 10^{-4}$ and the prior probability of a variant associated with both traits is $1\times 10^{-5}$.

## Data and code availability

The GWAS summary statistics used in this study are publicly available at the URLs below. UK Biobank, https://www.nealelab.is/uk-biobank; FinnGen R9, https://finngen.gitbook.io/documentation/v/r9/data-download. The proposed framework is implemented in R, which is publicly available at https://github.com/ZhaotongL/CMR.

## Supplemental information

Document S1. Figures S1–S7 and Tables S1–S20

Document S2. Article plus supplemental information

## References

[1] Sanderson E., Glymour M.M., Holmes M.V., Kang H., Morrison J., Munafò M.R., Palmer T., Schooling C.M., Wallace C., Zhao Q., Smith G.D. (2022). Mendelian randomization. Nat. Rev. Methods Primers.

[2] Staley J.R., Blackshaw J., Kamat M.A., Ellis S., Surendran P., Sun B.B., Paul D.S., Freitag D., Burgess S., Danesh J. (2016). Phenoscanner: a database of human genotype-phenotype associations. Bioinformatics.

[3] Hemani G., Tilling K., Davey Smith G. (2017). Orienting the causal relationship between imprecisely measured traits using gwas summary data. PLoS Genet..

[4] Burgess S., Thompson S.G. (2015). Multivariable mendelian randomization: the use of pleiotropic genetic variants to estimate causal effects. Am. J. Epidemiol..

[5] Bowden J., Del Greco M F., Minelli C., Davey Smith G., Sheehan N., Thompson J. (2017). A framework for the investigation of pleiotropy in two-sample summary data mendelian randomization. Stat. Med..

[6] Bowden J., Davey Smith G., Burgess S. (2015). Mendelian randomization with invalid instruments: effect estimation and bias detection through egger regression. Int. J. Epidemiol..

[7] Zhao Q., Wang J., Hemani G., Bowden J., Small D.S. (2020). Statistical inference in two-sample summary-data mendelian randomization using robust adjusted profile score. Ann. Stat..

[8] Bowden J., Davey Smith G., Haycock P.C., Burgess S. (2016). Consistent estimation in mendelian randomization with some invalid instruments using a weighted median estimator. Genet. Epidemiol..

[9] Hartwig F.P., Davey Smith G., Bowden J. (2017). Robust inference in summary data mendelian randomization via the zero modal pleiotropy assumption. Int. J. Epidemiol..

[10] Xue H., Shen X., Pan W. (2021). Constrained maximum likelihood-based mendelian randomization robust to both correlated and uncorrelated pleiotropic effects. Am. J. Hum. Genet..

[11] Burgess S., Smith G.D., Davies N.M., Dudbridge F., Gill D., Glymour M.M., Hartwig F.P., Kutalik Z., Holmes M.V., Minelli C. (2019). Guidelines for performing mendelian randomization investigations: update for summer 2023. Wellcome Open Res..

[12] Fortune M.D., Guo H., Burren O., Schofield E., Walker N.M., Ban M., Sawcer S.J., Bowes J., Worthington J., Barton A. (2015). Statistical colocalization of genetic risk variants for related autoimmune diseases in the context of common controls. Nat. Genet..

[13] Pickrell J.K., Berisa T., Liu J.Z., Ségurel L., Tung J.Y., Hinds D.A. (2016). Detection and interpretation of shared genetic influences on 42 human traits. Nat. Genet..

[14] van der Graaf A., Claringbould A., Rimbert A., Westra H.J., Li Y., Wijmenga C., Sanna S., BIOS Consortium (2020). Mendelian randomization while jointly modeling cis genetics identifies causal relationships between gene expression and lipids. Nat. Commun..

[15] Yang J., Ferreira T., Morris A.P., Medland S.E., Madden P.A.F., Heath A.C., Martin N.G., Montgomery G.W., Genetic Investigation of ANthropometric Traits GIANT Consortium, DIAbetes Genetics Replication And Meta-analysis DIAGRAM Consortium (2012). Conditional and joint multiple-snp analysis of gwas summary statistics identifies additional variants influencing complex traits. Nat. Genet..

[16] Sanderson E., Richardson T.G., Hemani G., Davey Smith G. (2021). The use of negative control outcomes in mendelian randomization to detect potential population stratification. Int. J. Epidemiol..

[17] Lawlor D.A., Harbord R.M., Sterne J.A.C., Timpson N., Davey Smith G. (2008). Mendelian randomization: using genes as instruments for making causal inferences in epidemiology. Stat. Med..

[18] Bowden J., Del Greco M F., Minelli C., Zhao Q., Lawlor D.A., Sheehan N.A., Thompson J., Davey Smith G. (2019). Improving the accuracy of two-sample summary-data mendelian randomization: moving beyond the nome assumption. Int. J. Epidemiol..

[19] Powell-Wiley T.M., Poirier P., Burke L.E., Després J.-P., Gordon-Larsen P., Lavie C.J., Lear S.A., Ndumele C.E., Neeland I.J., Sanders P. (2021). Obesity and cardiovascular disease: a scientific statement from the american heart association. Circulation.

[20] Burgess S., Smith G.D., Davies N.M., Dudbridge F., Gill D., Glymour M.M., Hartwig F.P., Kutalik Z., Holmes M.V., Minelli C. (2023). Guidelines for performing mendelian randomization investigations: update for summer 2023 [version 3; peer review: 2 approved]. Wellcome Open Res..

[21] Giambartolomei C., Vukcevic D., Schadt E.E., Franke L., Hingorani A.D., Wallace C., Plagnol V. (2014). Bayesian test for colocalisation between pairs of genetic association studies using summary statistics. PLoS Genet..

[22] Hemani G., Bowden J., Davey Smith G. (2018). Evaluating the potential role of pleiotropy in mendelian randomization studies. Hum. Mol. Genet..

[23] Cho Y., Haycock P.C., Sanderson E., Gaunt T.R., Zheng J., Morris A.P., Davey Smith G., Hemani G. (2020). Exploiting horizontal pleiotropy to search for causal pathways within a mendelian randomization framework. Nat. Commun..

[24] Hu X., Zhao J., Lin Z., Wang Y., Peng H., Zhao H., Wan X., Yang C. (2022). Mendelian randomization for causal inference accounting for pleiotropy and sample structure using genome-wide summary statistics. Proc. Natl. Acad. Sci. USA.

[25] Lin Z., Xue H., Pan W. (2023). Combining mendelian randomization and network deconvolution for inference of causal networks with gwas summary data. PLoS Genet..

[26] Cheng Q., Yang Y., Shi X., Yeung K.-F., Yang C., Peng H., Liu J. (2020). Mr-ldp: a two-sample mendelian randomization for gwas summary statistics accounting for linkage disequilibrium and horizontal pleiotropy. NAR Genom. Bioinform..

[27] Cheng Q., Zhang X., Chen L.S., Liu J. (2022). Mendelian randomization accounting for complex correlated horizontal pleiotropy while elucidating shared genetic etiology. Nat. Commun..

[28] Burgess S., Davies N.M., Thompson S.G. (2016). Bias due to participant overlap in two-sample mendelian randomization. Genet. Epidemiol..

[29] Berisa T., Pickrell J.K. (2016). Approximately independent linkage disequilibrium blocks in human populations. Bioinformatics.

[30] Chang C.C., Chow C.C., Tellier L.C.A.M., Vattikuti S., Purcell S.M., Lee J.J. (2015). Second-generation plink: rising to the challenge of larger and richer datasets. GigaScience.

[31] Kurki M.I., Karjalainen J., Palta P., Sipilä T.P., Kristiansson K., Donner K.M., Reeve M.P., Laivuori H., Aavikko M., Kaunisto M.A. (2023). Finngen provides genetic insights from a well-phenotyped isolated population. Nature.

[32] Bycroft C., Freeman C., Petkova D., Band G., Elliott L.T., Sharp K., Motyer A., Vukcevic D., Delaneau O., O’Connell J. (2017). Genome-wide genetic data on∼ 500,000 uk biobank participants. bioRxiv.

[33] Hemani G., Zheng J., Elsworth B., Wade K.H., Haberland V., Baird D., Laurin C., Burgess S., Bowden J., Langdon R. (2018). The mr-base platform supports systematic causal inference across the human phenome. Elife.

[34] Patel A., Ye T., Xue H., Lin Z., Xu S., Woolf B., Mason A.M., Burgess S. (2023). Mendelianrandomization v0. 9.0: updates to an r package for performing mendelian randomization analyses using summarized data. Wellcome Open Res..

