# Supplementary material for: A novel framework with automated horizontal pleiotropy adjustment in mendelian randomization
Source: HGG Adv. 2024 Aug 2;5(4):100339. doi: 10.1016/j.xhgg.2024.100339 (PMC11375132; doi:10.1016/j.xhgg.2024.100339)
Supplement: Document S1. Figures S1–S7 and Tables S1–S20 [file mmc1.pdf]

HGGA, Volume *5*

Supplemental information

A novel framework with automated horizontal pleiotropy adjustment in mendelian randomization

Zhaotong Lin

Supplemental Figures

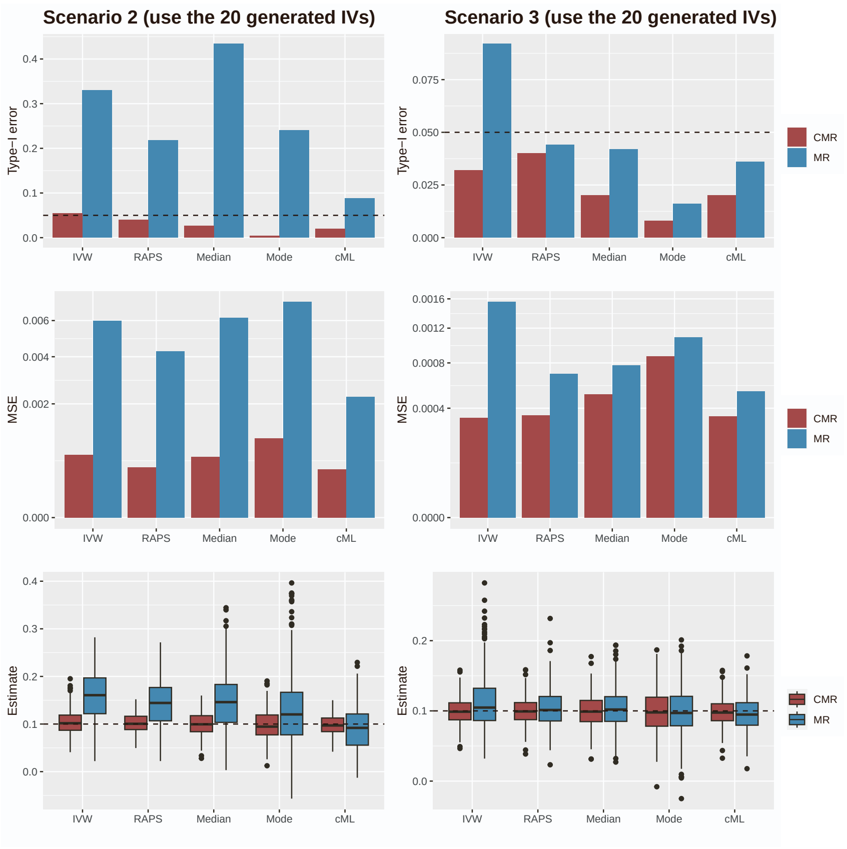

Figure S1. **Addtional simulation results for Scenarios 2 (left) and 3 (right).** Here we directly used the 20 generated IVs Z_j in both the standard MR and CMR implementations without selection of IVs via LD clumping. From top to bottom are type-I errors, mean squared error (MSE) and boxplots of estimates across 500 replicates.

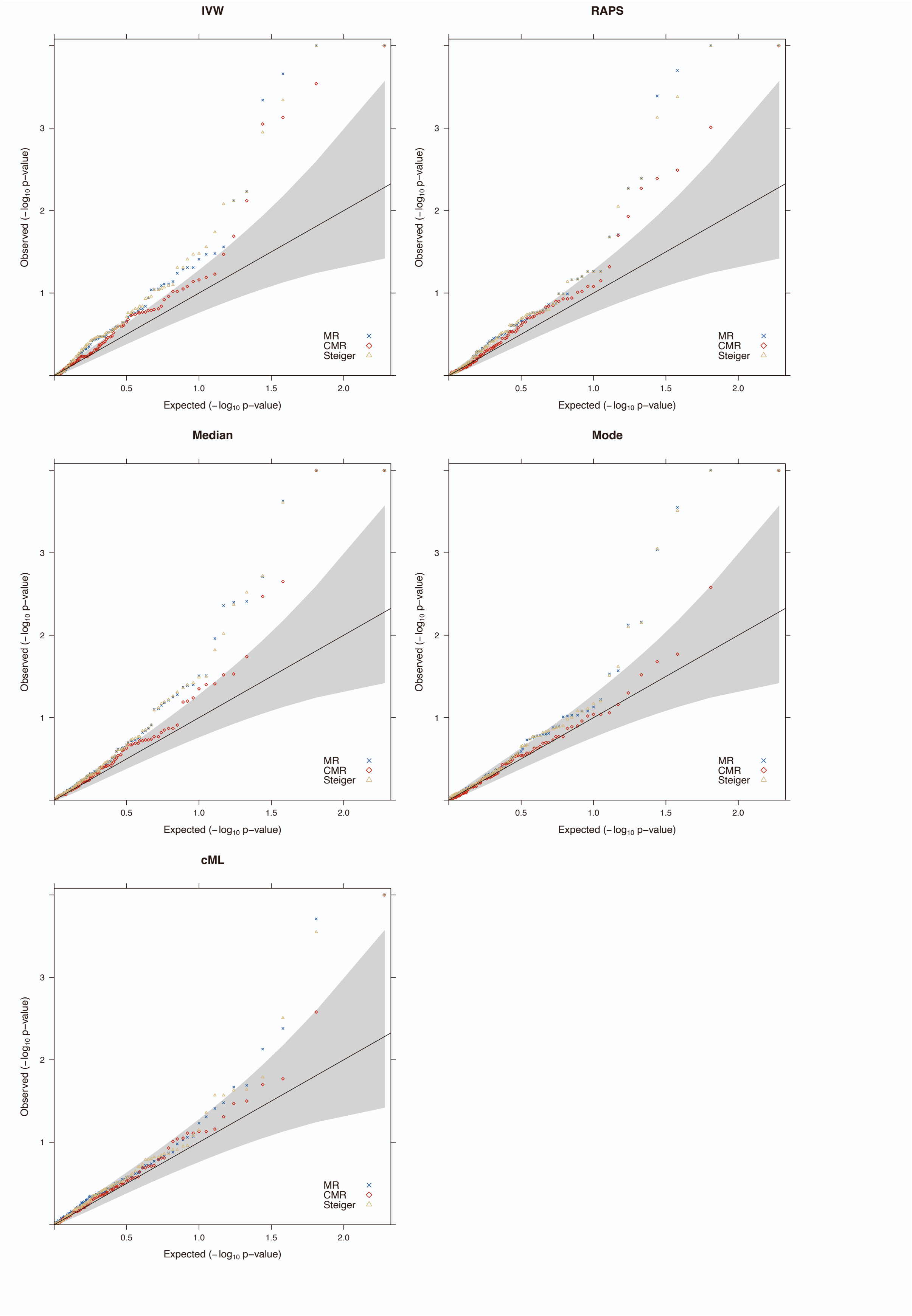

Figure S2. **Q-Q plots of the 96 pairs negative control analysis.** Within each plot, 'MR' is applying the standard MR method alone, 'Steiger' is applying the standard MR method after Steiger filtering, and 'CMR' is implementing the proposed CMR framework.

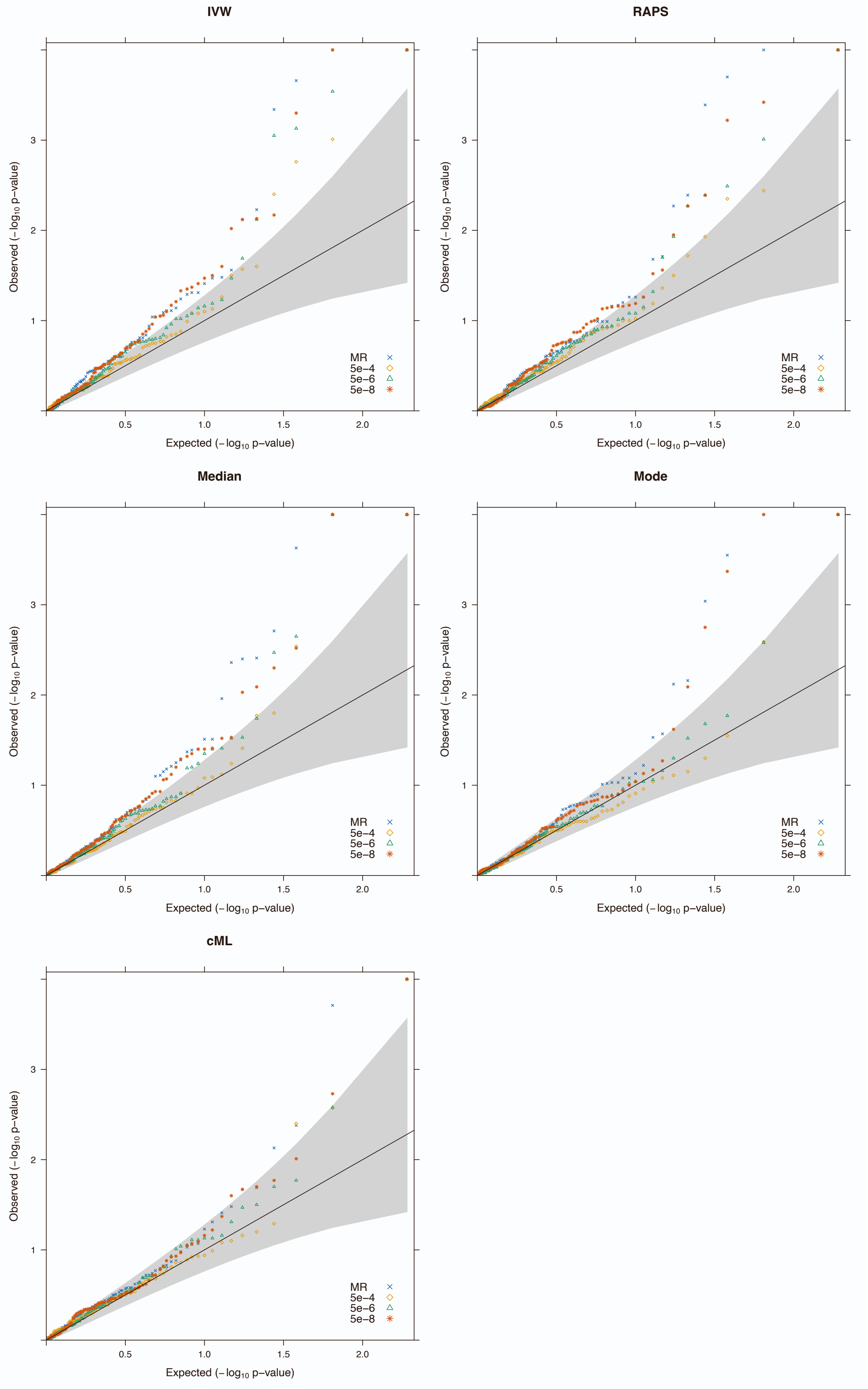

Figure S3. **Q-Q plots of the 96 pairs negative control analysis with different COJO p-value thresholds.** Within each plot, 'MR' is applying the standard MR method alone, '5e-4','5e-6' and '5e-8' correspond to applying the CMR framework with different p-value thresholds to select outcome-associated SNPs.

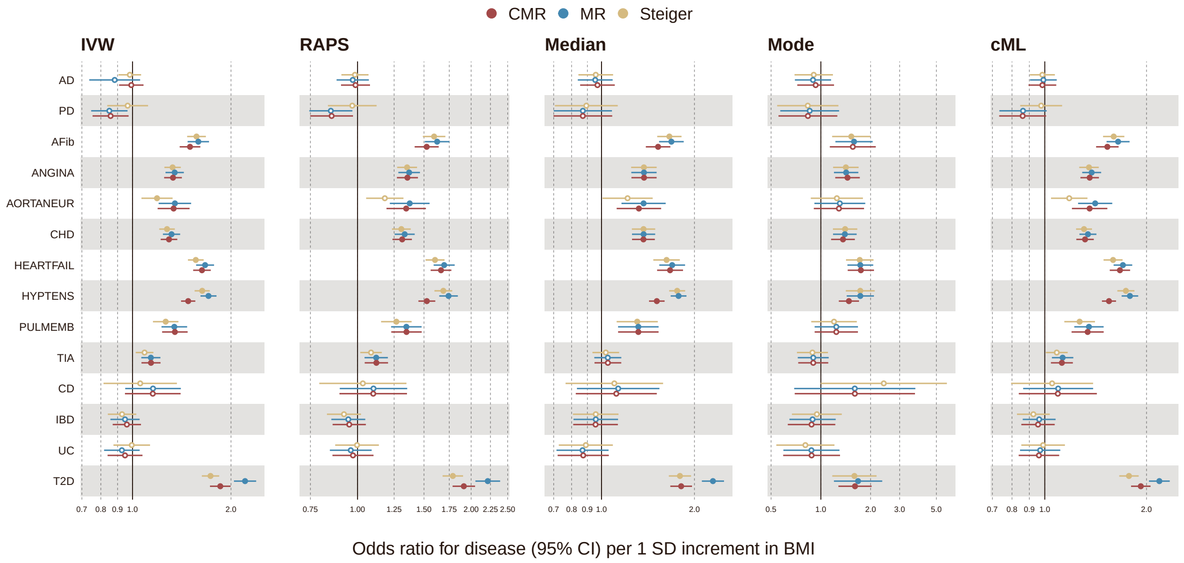

Figure S4. **Forest plots of causal effect estimates (X-axis) of BMI on multiple diseases (Y-axis) obtained from different methods in both the standard MR (with and without Steiger filtering) and CMR frameworks.** Solid circles represent statistically significant results after Bonferroni correction with $p \leq 0.05/14$. 'MR' is applying the standard MR method alone, 'Steiger' is applying the standard MR method after Steiger filtering, and 'CMR' is implementing the proposed CMR framework.

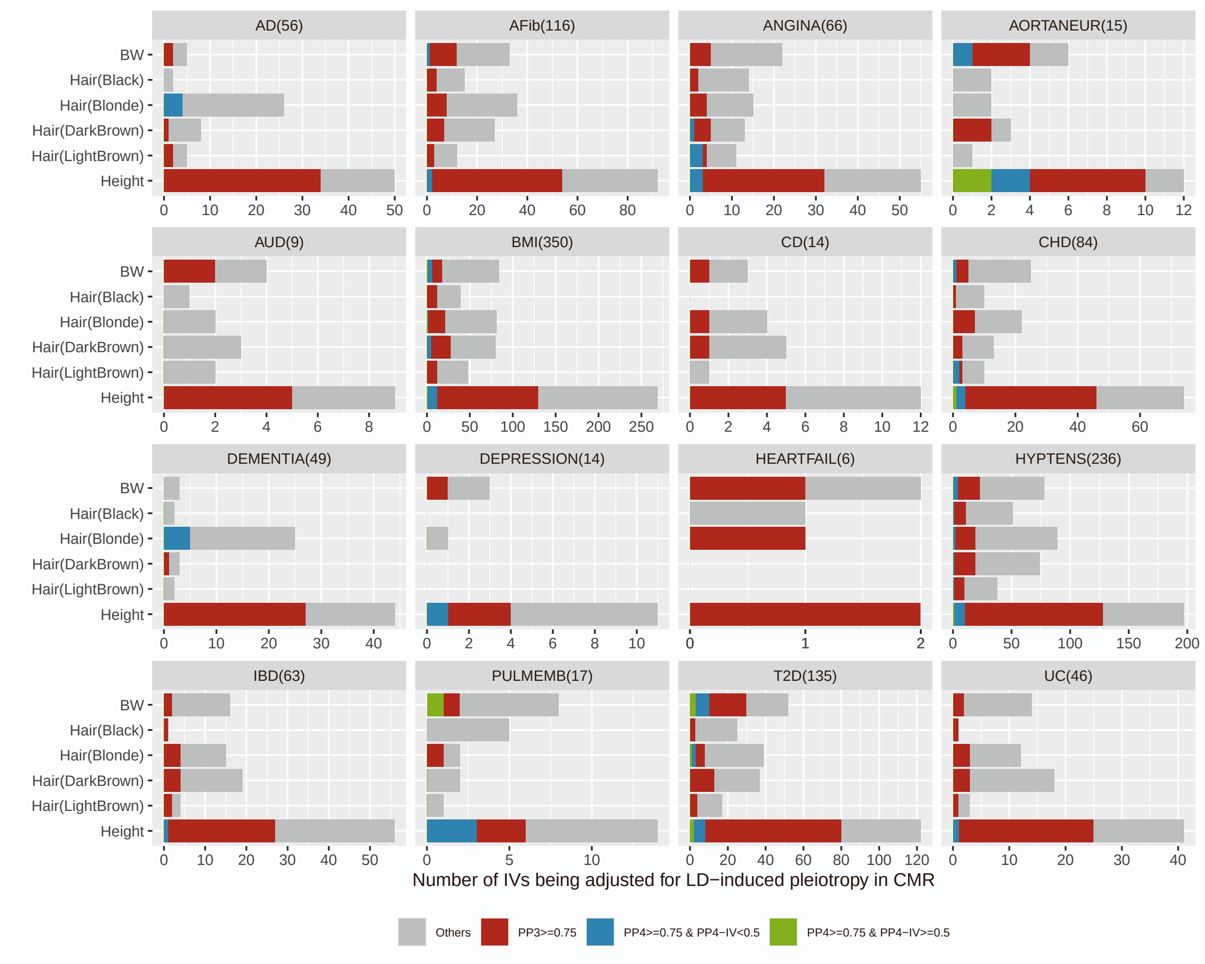

Figure S5. **Number of IVs being adjusted for LD-induced pleiotropy in negative control outcome analysis.** Colors indicate colocalization results. Numbers in parentheses are the total number of IVs used in the MR analysis. AD, Alzheimer's disease; AFib, atrial fibrillation; ANGINA, angina pectoris; AORTANEUR, aortic aneurysm; AUD, alcohol use disorder; BMI, body mass index; CHD, coronary heart disease; HEARTFAIL, heart failure; HYPTENS, hypertension; PULMEMB, pulmonary embolism; TIA, transient ischemic attack; CD, Crohn disease; IBD, inflammatory bowel disease; UC, ulcerative colitis; T2D, type-II diabetes; BW: birth weight.

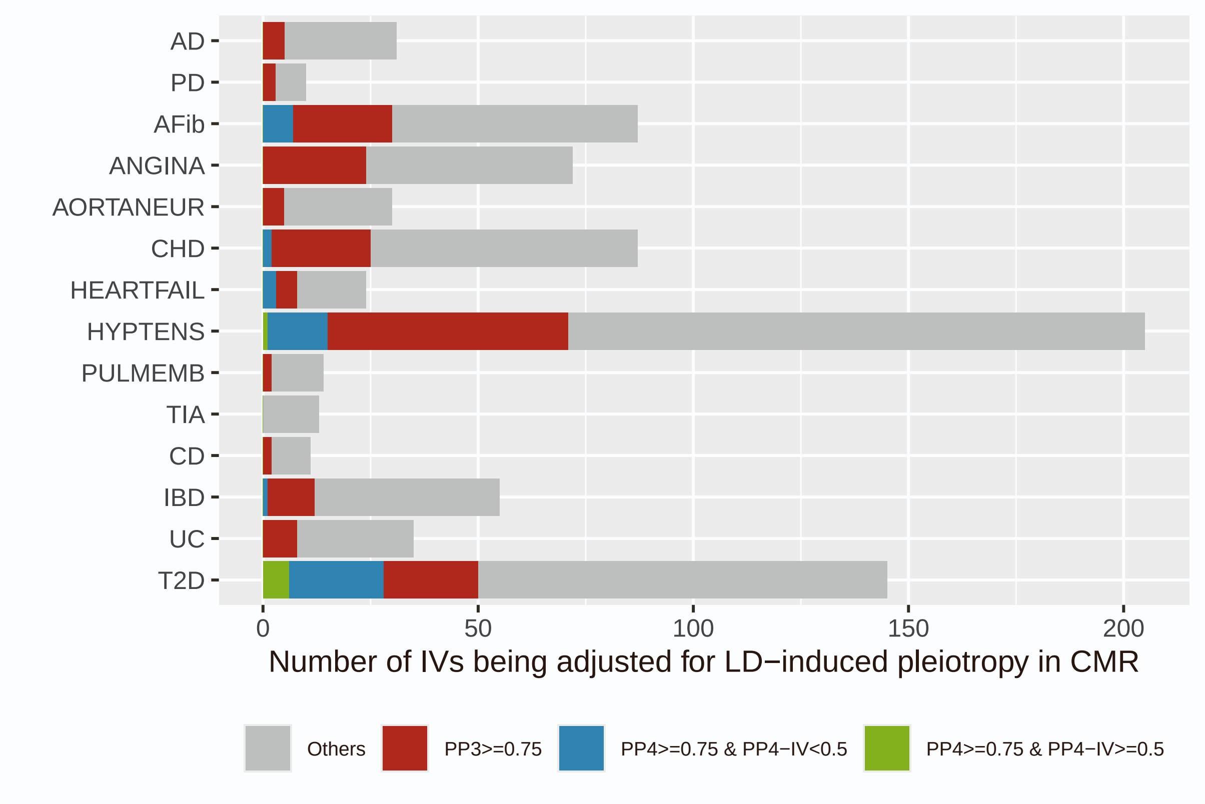

Figure S6. **Number of IVs being adjusted for LD-induced pleiotropy in the investigation of the role of BMI in multiple diseases.** Colors indicate colocalization results. The total number of IVs for BMI is 453. AD, Alzheimer's disease; PD, Parkinson's disease; AFib, atrial fibrillation; ANGINA, angina pectoris; AORTANEUR, aortic aneurysm; CHD, coronary heart disease; HEARTFAIL, heart failure; HYPTENS, hypertension; PULMEMB, pulmonary embolism; TIA, transient ischemic attack; CD, Crohn disease; IBD, inflammatory bowel disease; UC, ulcerative colitis; T2D, type-II diabetes.

Supplemental Tables

	$\theta = 0$					$\theta = 0.05$					$\theta = 0.1$				
	mean($\hat{\theta}$)	MSE	T1E	SD	SE	mean($\hat{\theta}$)	MSE	Power	SD	SE	mean($\hat{\theta}$)	MSE	Power	SD	SE
**MR**															
IVW	0.083	1.78E-02	0.118	0.104	0.098	0.133	1.77E-02	0.260	0.105	0.098	0.182	1.77E-02	0.488	0.105	0.098
Median	0.021	1.07E-02	0.416	0.102	0.040	0.070	1.08E-02	0.500	0.102	0.040	0.120	1.10E-02	0.668	0.103	0.041
Mode	-0.014	1.25E-02	0.398	0.111	0.051	0.027	1.23E-02	0.384	0.108	0.052	0.072	1.26E-02	0.426	0.109	0.053
RAPS	0.053	1.18E-02	0.090	0.095	0.092	0.103	1.18E-02	0.182	0.095	0.092	0.153	1.18E-02	0.362	0.095	0.092
cML	-0.020	4.71E-03	0.132	0.066	0.054	0.008	6.01E-03	0.108	0.065	0.053	0.038	8.67E-03	0.152	0.070	0.054
CUE	0.083	1.85E-02	0.106	0.108	0.106	0.132	1.75E-02	0.210	0.103	0.106	0.186	1.84E-02	0.400	0.105	0.107
APSS	0.088	1.80E-02	0.110	0.101	0.099	0.138	1.79E-02	0.216	0.101	0.101	0.187	1.79E-02	0.432	0.102	0.103
MIX	-0.055	4.21E-02	0.566	0.198	0.063	-0.005	4.09E-02	0.484	0.195	0.062	0.039	3.61E-02	0.470	0.180	0.062
AID	-0.016	2.02E-02	0.478	0.141		0.036	2.03E-02	0.448	0.142		0.080	1.94E-02	0.462	0.138	
**CMR**															
IVW	0.017	1.64E-03	0.056	0.037	0.032	0.065	1.63E-03	0.546	0.037	0.032	0.114	1.63E-03	0.960	0.038	0.032
Median	0.002	7.76E-04	0.048	0.028	0.028	0.051	8.32E-04	0.430	0.029	0.029	0.100	8.96E-04	0.908	0.030	0.030
Mode	-0.001	1.15E-03	0.016	0.034	0.040	0.048	1.25E-03	0.198	0.035	0.041	0.096	1.37E-03	0.648	0.037	0.042
RAPS	0.005	5.80E-04	0.054	0.024	0.025	0.054	6.10E-04	0.562	0.024	0.026	0.103	6.52E-04	0.964	0.025	0.026
cML	0.001	4.47E-04	0.040	0.021	0.022	0.048	5.12E-04	0.530	0.023	0.023	0.098	5.69E-04	0.972	0.024	0.024
CUE	0.008	7.98E-04	0.048	0.027	0.028	0.058	9.25E-04	0.564	0.029	0.029	0.107	9.53E-04	0.956	0.030	0.029
APSS	0.019	1.83E-03	0.050	0.038	0.032	0.068	1.80E-03	0.550	0.039	0.034	0.117	1.80E-03	0.948	0.039	0.037
MIX	-0.001	1.04E-03	0.034	0.032	0.051	0.043	1.20E-03	0.330	0.034	0.051	0.086	1.61E-03	0.714	0.038	0.081
AID	0.001	4.41E-04	0.058	0.021		0.047	4.66E-04	0.654	0.021		0.091	5.48E-04	0.988	0.022	

Average F-statistics when $\theta = 0$ across 500 replicates before and after conditioning are 168 and 144.

Table S1. Simulation results for Scenario 1 with 0% IVs having *biological* horizontal pleiotropy across 500 replicates. From left to right: causal effect $\theta = 0, 0.05, 0.1$. The top part corresponds to results from standard MR methods and the bottom part corresponds to results from the CMR implementation. MSE: mean squared error. T1E: type-1 error. SD: standard deviation of estimates. SE: mean of estimated standard error.

	$\theta = 0$					$\theta = 0.05$					$\theta = 0.1$				
	mean($\hat{\theta}$)	MSE	T1E	SD	SE	mean($\hat{\theta}$)	MSE	Power	SD	SE	mean($\hat{\theta}$)	MSE	Power	SD	SE
**MR**															
IVW	0.086	1.99E-02	0.130	0.112	0.106	0.136	1.98E-02	0.250	0.112	0.106	0.185	1.98E-02	0.406	0.112	0.106
Median	0.031	1.40E-02	0.470	0.114	0.041	0.080	1.40E-02	0.536	0.115	0.042	0.131	1.40E-02	0.666	0.114	0.043
Mode	-0.007	1.60E-02	0.442	0.126	0.053	0.036	1.64E-02	0.410	0.127	0.054	0.079	1.70E-02	0.458	0.129	0.055
RAPS	0.061	1.45E-02	0.106	0.104	0.101	0.111	1.45E-02	0.188	0.104	0.101	0.161	1.45E-02	0.332	0.104	0.102
cML	-0.015	6.51E-03	0.148	0.079	0.059	0.011	7.83E-03	0.110	0.080	0.059	0.039	1.07E-02	0.156	0.084	0.060
CUE	0.088	2.02E-02	0.098	0.111	0.115	0.138	1.99E-02	0.204	0.111	0.115	0.189	2.04E-02	0.338	0.112	0.114
APSS	0.091	1.98E-02	0.116	0.108	0.106	0.141	1.98E-02	0.218	0.108	0.109	0.190	1.98E-02	0.354	0.108	0.111
MIX	-0.052	4.80E-02	0.620	0.213	0.071	-0.005	4.63E-02	0.554	0.208	0.069	0.040	4.57E-02	0.510	0.205	0.067
AID	-0.002	3.16E-02	0.552	0.178		0.040	2.76E-02	0.486	0.166		0.090	2.71E-02	0.508	0.164	
**CMR**															
IVW	0.020	3.64E-03	0.064	0.057	0.050	0.069	3.66E-03	0.290	0.057	0.050	0.118	3.70E-03	0.674	0.058	0.050
Median	0.002	9.95E-04	0.058	0.031	0.031	0.051	1.01E-03	0.386	0.032	0.031	0.100	1.08E-03	0.872	0.033	0.032
Mode	-0.001	1.05E-03	0.026	0.032	0.039	0.047	1.09E-03	0.178	0.033	0.040	0.096	1.17E-03	0.674	0.034	0.041
RAPS	0.010	1.42E-03	0.044	0.036	0.040	0.059	1.41E-03	0.302	0.036	0.040	0.108	1.44E-03	0.762	0.037	0.041
cML	0.000	5.80E-04	0.032	0.024	0.026	0.047	6.87E-04	0.396	0.026	0.028	0.094	8.25E-04	0.866	0.028	0.030
CUE	0.015	2.25E-03	0.054	0.045	0.047	0.063	2.10E-03	0.272	0.044	0.047	0.112	2.27E-03	0.672	0.046	0.048
APSS	0.023	3.39E-03	0.046	0.053	0.051	0.072	3.37E-03	0.244	0.054	0.052	0.120	3.35E-03	0.626	0.054	0.055
MIX	0.000	1.08E-03	0.036	0.033	0.044	0.047	1.07E-03	0.262	0.033	0.041	0.091	1.15E-03	0.676	0.033	0.042
AID	0.001	6.86E-04	0.098	0.026		0.048	6.84E-04	0.552	0.026		0.092	7.54E-04	0.962	0.026	

Average F-statistics when $\theta = 0$ across 500 replicates before and after conditioning are 168 and 143.

Table S2. Simulation results for Scenario 1 with 20% IVs having *biological* horizontal pleiotropy across 500 replicates. From left to right: causal effect $\theta = 0, 0.05, 0.1$. The top part corresponds to results from standard MR methods and the bottom part corresponds to results from the CMR implementation. MSE: mean squared error. T1E: type-1 error. SD: standard deviation of estimates. SE: mean of estimated standard error.

	$\theta = 0$					$\theta = 0.05$					$\theta = 0.1$				
	mean($\hat{\theta}$)	MSE	T1E	SD	SE	mean($\hat{\theta}$)	MSE	Power	SD	SE	mean($\hat{\theta}$)	MSE	Power	SD	SE
**MR**															
IVW	0.088	2.24E-02	0.134	0.121	0.113	0.137	2.24E-02	0.236	0.122	0.113	0.187	2.24E-02	0.374	0.122	0.113
Median	0.042	1.93E-02	0.542	0.133	0.043	0.092	1.94E-02	0.578	0.133	0.043	0.142	1.96E-02	0.684	0.134	0.044
Mode	0.005	2.02E-02	0.500	0.142	0.053	0.048	2.03E-02	0.472	0.143	0.055	0.090	2.06E-02	0.496	0.143	0.057
RAPS	0.066	1.75E-02	0.104	0.115	0.110	0.116	1.75E-02	0.166	0.115	0.110	0.166	1.75E-02	0.336	0.115	0.110
cML	-0.008	7.39E-03	0.112	0.086	0.063	0.016	8.82E-03	0.126	0.088	0.063	0.042	1.24E-02	0.158	0.095	0.064
CUE	0.089	2.30E-02	0.102	0.123	0.122	0.141	2.34E-02	0.176	0.123	0.123	0.189	2.32E-02	0.344	0.123	0.122
APSS	0.092	2.20E-02	0.094	0.117	0.113	0.142	2.20E-02	0.198	0.117	0.115	0.191	2.21E-02	0.378	0.117	0.117
MIX	-0.016	6.19E-02	0.624	0.249	0.070	0.030	6.08E-02	0.558	0.246	0.072	0.075	5.68E-02	0.562	0.237	0.068
AID	0.020	4.28E-02	0.546	0.206		0.061	3.84E-02	0.522	0.196		0.117	3.82E-02	0.542	0.195	
**CMR**															
IVW	0.022	5.63E-03	0.112	0.072	0.063	0.070	5.60E-03	0.240	0.072	0.063	0.118	5.64E-03	0.468	0.073	0.063
Median	0.005	1.76E-03	0.104	0.042	0.034	0.054	1.85E-03	0.374	0.043	0.034	0.102	1.95E-03	0.786	0.044	0.035
Mode	0.000	1.46E-03	0.026	0.038	0.041	0.048	1.53E-03	0.224	0.039	0.042	0.096	1.62E-03	0.636	0.040	0.044
RAPS	0.014	3.70E-03	0.078	0.059	0.056	0.063	3.66E-03	0.224	0.059	0.056	0.111	3.75E-03	0.510	0.060	0.057
cML	0.002	1.04E-03	0.054	0.032	0.031	0.046	1.20E-03	0.274	0.034	0.033	0.091	1.52E-03	0.664	0.038	0.036
CUE	0.023	5.02E-03	0.064	0.067	0.068	0.072	5.29E-03	0.196	0.069	0.068	0.119	4.94E-03	0.432	0.068	0.067
APSS	0.025	5.30E-03	0.086	0.069	0.064	0.073	5.24E-03	0.220	0.069	0.065	0.121	5.25E-03	0.420	0.069	0.067
MIX	0.001	2.05E-03	0.076	0.045	0.044	0.048	1.93E-03	0.274	0.044	0.045	0.093	2.04E-03	0.626	0.045	0.046
AID	0.004	1.63E-03	0.152	0.040		0.049	1.48E-03	0.484	0.039		0.094	1.58E-03	0.864	0.039	

Average F-statistics when $\theta = 0$ across 500 replicates before and after conditioning are 168 and 142.

Table S3. Simulation results for Scenario 1 with 40% IVs having *biological* horizontal pleiotropy across 500 replicates. From left to right: causal effect $\theta = 0, 0.05, 0.1$. The top part corresponds to results from standard MR methods and the bottom part corresponds to results from the CMR implementation. MSE: mean squared error. T1E: type-1 error. SD: standard deviation of estimates. SE: mean of estimated standard error.

	$\theta = 0$					$\theta = 0.05$					$\theta = 0.1$				
	mean($\hat{\theta}$)	MSE	T1E	SD	SE	mean($\hat{\theta}$)	MSE	Power	SD	SE	mean($\hat{\theta}$)	MSE	Power	SD	SE
**MR**															
IVW	0.088	0.025	0.136	0.131	0.118	0.138	0.025	0.234	0.131	0.118	0.187	0.025	0.358	0.131	0.119
Median	0.045	0.024	0.558	0.147	0.044	0.094	0.024	0.620	0.148	0.045	0.144	0.024	0.684	0.148	0.046
Mode	0.011	0.026	0.544	0.161	0.059	0.047	0.025	0.520	0.158	0.060	0.086	0.025	0.536	0.157	0.062
RAPS	0.067	0.019	0.100	0.121	0.116	0.117	0.019	0.176	0.121	0.116	0.167	0.019	0.294	0.121	0.116
cML	-0.012	0.009	0.136	0.093	0.068	0.009	0.010	0.130	0.093	0.067	0.032	0.014	0.146	0.096	0.068
CUE	0.091	0.024	0.090	0.126	0.128	0.142	0.024	0.182	0.127	0.128	0.192	0.024	0.296	0.126	0.128
APSS	0.092	0.024	0.110	0.123	0.119	0.142	0.024	0.202	0.123	0.121	0.191	0.024	0.314	0.123	0.122
MIX	-0.036	0.076	0.652	0.273	0.065	0.018	0.073	0.616	0.269	0.068	0.064	0.070	0.626	0.263	0.066
AID	0.021	0.045	0.562	0.211		0.072	0.045	0.562	0.210		0.117	0.040	0.558	0.201	
**CMR**															
IVW	0.025	0.008	0.112	0.084	0.072	0.073	0.008	0.216	0.084	0.072	0.121	0.008	0.394	0.084	0.072
Median	0.010	0.004	0.186	0.060	0.037	0.058	0.004	0.392	0.061	0.038	0.106	0.004	0.706	0.062	0.039
Mode	0.003	0.004	0.100	0.061	0.048	0.050	0.004	0.242	0.062	0.049	0.097	0.004	0.548	0.060	0.051
RAPS	0.019	0.006	0.096	0.075	0.069	0.066	0.006	0.202	0.076	0.069	0.114	0.006	0.388	0.077	0.070
cML	0.003	0.002	0.062	0.042	0.039	0.043	0.002	0.168	0.045	0.042	0.082	0.003	0.414	0.051	0.046
CUE	0.027	0.007	0.070	0.081	0.078	0.075	0.007	0.176	0.079	0.078	0.123	0.007	0.346	0.079	0.078
APSS	0.028	0.007	0.080	0.079	0.073	0.076	0.007	0.176	0.079	0.074	0.123	0.007	0.354	0.079	0.076
MIX	-0.003	0.007	0.140	0.086	0.057	0.044	0.008	0.256	0.087	0.056	0.085	0.009	0.522	0.096	0.055
AID	0.004	0.004	0.210	0.061		0.051	0.004	0.402	0.061		0.096	0.004	0.728	0.064	

Average F-statistics when $\theta = 0$ across 500 replicates before and after conditioning are 168 and 142.

Table S4. Simulation results for Scenario 1 with 60% IVs having *biological* horizontal pleiotropy across 500 replicates. From left to right: causal effect $\theta = 0, 0.05, 0.1$. The top part corresponds to results from standard MR methods and the bottom part corresponds to results from the CMR implementation. MSE: mean squared error. T1E: type-1 error. SD: standard deviation of estimates. SE: mean of estimated standard error.

	$\theta = 0$					$\theta = 0.05$					$\theta = 0.1$				
	mean($\hat{\theta}$)	MSE	T1E	SD	SE	mean($\hat{\theta}$)	MSE	Power	SD	SE	mean($\hat{\theta}$)	MSE	Power	SD	SE
**MR**															
IVW	0.222	5.46E-02	0.868	0.074	0.080	0.271	5.43E-02	0.972	0.074	0.081	0.320	5.39E-02	0.996	0.074	0.081
Median	0.100	1.62E-02	0.660	0.079	0.033	0.150	1.63E-02	0.892	0.079	0.034	0.201	1.64E-02	0.984	0.080	0.035
Mode	0.036	5.16E-03	0.330	0.062	0.034	0.084	5.01E-03	0.562	0.062	0.035	0.132	4.99E-03	0.864	0.063	0.035
RAPS	0.188	4.15E-02	0.670	0.079	0.080	0.237	4.13E-02	0.928	0.079	0.080	0.287	4.12E-02	0.986	0.079	0.080
cML	0.007	1.46E-03	0.090	0.038	0.031	0.050	1.75E-03	0.360	0.042	0.032	0.099	2.03E-03	0.746	0.045	0.034
CUE	0.201	5.34E-02	0.658	0.114	0.085	0.251	5.31E-02	0.856	0.112	0.086	0.303	5.38E-02	0.958	0.113	0.084
MIX	0.015	2.57E-02	0.212	0.160	0.046	0.064	2.20E-02	0.294	0.148	0.045	0.109	1.86E-02	0.556	0.136	0.050
AID	0.032	2.75E-02	0.240	0.163		0.076	1.95E-02	0.484	0.137		0.125	1.99E-02	0.800	0.139	
**CMR**															
IVW	0.183	4.01E-02	0.628	0.081	0.080	0.232	3.97E-02	0.928	0.081	0.081	0.281	3.92E-02	0.996	0.082	0.081
Median	0.027	2.80E-03	0.128	0.046	0.028	0.076	3.03E-03	0.734	0.048	0.029	0.125	2.96E-03	0.992	0.048	0.030
Mode	0.001	8.02E-04	0.014	0.028	0.040	0.049	9.52E-04	0.378	0.031	0.042	0.097	8.74E-04	0.882	0.029	0.043
RAPS	0.118	2.13E-02	0.256	0.086	0.069	0.166	2.08E-02	0.688	0.086	0.069	0.215	2.06E-02	0.982	0.086	0.070
cML	0.001	3.43E-04	0.026	0.019	0.021	0.048	3.78E-04	0.588	0.019	0.022	0.097	4.17E-04	0.992	0.020	0.023
CUE	0.108	5.26E-02	0.252	0.203	0.059	0.153	4.99E-02	0.698	0.199	0.058	0.206	4.89E-02	0.932	0.194	0.062
MIX	-0.001	5.19E-04	0.020	0.023	0.029	0.047	5.17E-04	0.400	0.023	0.029	0.092	5.77E-04	0.920	0.023	0.030
AID	0.008	5.12E-03	0.044	0.071		0.060	9.41E-03	0.682	0.097		0.102	4.81E-03	0.994	0.069	

Average F-statistics when $\theta = 0$ across 500 replicates before and after conditioning are 175 and 150.

Table S5. Simulation results for Scenario 2 across 500 replicates. From left to right: causal effect $\theta = 0, 0.05, 0.1$. The top part corresponds to results from standard MR methods and the bottom part corresponds to results from the CMR implementation. MSE: mean squared error. T1E: type-1 error. SD: standard deviation of estimates. SE: mean of estimated standard error.

	$\theta = 0$					$\theta = 0.05$					$\theta = 0.1$				
	mean($\hat{\theta}$)	MSE	T1E	SD	SE	mean($\hat{\theta}$)	MSE	Power	SD	SE	mean($\hat{\theta}$)	MSE	Power	SD	SE
**MR**															
IVW	0.172	3.43E-02	0.496	0.068	0.086	0.221	3.40E-02	0.860	0.068	0.087	0.271	3.37E-02	0.976	0.068	0.087
Median	0.018	9.99E-04	0.100	0.026	0.025	0.068	1.03E-03	0.768	0.027	0.025	0.118	1.08E-03	0.992	0.028	0.026
Mode	0.001	6.44E-04	0.032	0.025	0.028	0.049	6.64E-04	0.418	0.026	0.028	0.098	7.20E-04	0.934	0.027	0.029
RAPS	0.094	1.19E-02	0.126	0.056	0.068	0.144	1.19E-02	0.590	0.056	0.068	0.193	1.18E-02	0.960	0.056	0.068
cML	-0.001	3.11E-04	0.032	0.018	0.019	0.047	3.75E-04	0.614	0.019	0.020	0.096	4.18E-04	0.992	0.020	0.021
CUE	0.115	5.52E-02	0.200	0.205	0.072	0.151	4.41E-02	0.708	0.184	0.067	0.212	5.01E-02	0.932	0.194	0.074
MIX	-0.004	6.14E-04	0.032	0.024	0.031	0.046	6.27E-04	0.354	0.025	0.030	0.093	6.60E-04	0.896	0.025	0.031
AID	0.000	3.74E-04	0.054	0.019		0.051	2.50E-03	0.750	0.050		0.096	4.01E-04	1.000	0.020	
**CMR**															
IVW	0.154	2.82E-02	0.374	0.066	0.083	0.204	2.80E-02	0.846	0.066	0.083	0.253	2.78E-02	0.984	0.067	0.083
Median	0.013	6.52E-04	0.058	0.022	0.024	0.063	6.66E-04	0.752	0.022	0.024	0.112	6.77E-04	0.996	0.023	0.025
Mode	0.002	5.39E-04	0.012	0.023	0.033	0.050	5.51E-04	0.396	0.023	0.034	0.099	5.72E-04	0.938	0.024	0.035
RAPS	0.071	7.39E-03	0.048	0.049	0.060	0.120	7.38E-03	0.502	0.049	0.060	0.170	7.32E-03	0.964	0.049	0.061
cML	0.000	2.43E-04	0.022	0.016	0.018	0.048	2.74E-04	0.756	0.016	0.019	0.098	2.92E-04	1.000	0.017	0.020
CUE	0.064	3.48E-02	0.146	0.175	0.047	0.103	2.56E-02	0.814	0.151	0.046	0.161	3.27E-02	0.960	0.170	0.050
MIX	-0.002	3.95E-04	0.018	0.020	0.027	0.047	4.08E-04	0.398	0.020	0.027	0.093	4.41E-04	0.956	0.020	0.027
AID	0.001	2.77E-04	0.028	0.017		0.050	2.75E-04	0.822	0.017		0.097	2.82E-04	1.000	0.017	

Average F-statistics when $\theta = 0$ across 500 replicates before and after conditioning are 148 and 144.

Table S6. Simulation results for Scenario 3 across 500 replicates. From left to right: causal effect $\theta = 0, 0.05, 0.1$. The top part corresponds to results from standard MR methods and the bottom part corresponds to results from the CMR implementation. MSE: mean squared error. T1E: type-1 error. SD: standard deviation of estimates. SE: mean of estimated standard error.

	$\theta = 0$					$\theta = 0.05$				
	mean($\hat{\theta}$)	MSE	T1E	SD	SE	mean($\hat{\theta}$)	MSE	Power	SD	SE
					**MR**					
IVW	-0.001	2.98E-04	0.044	0.017	0.018	0.049	3.17E-04	0.748	0.018	0.019
Median	-0.002	4.73E-04	0.026	0.022	0.024	0.048	5.00E-04	0.498	0.022	0.025
Mode	-0.002	8.03E-04	0.008	0.028	0.036	0.047	8.71E-04	0.206	0.029	0.037
RAPS	-0.001	3.15E-04	0.042	0.018	0.019	0.049	3.35E-04	0.724	0.018	0.019
cML	-0.001	2.89E-04	0.026	0.017	0.019	0.047	3.26E-04	0.682	0.018	0.020
					**CMR**					
IVW	-0.001	2.98E-04	0.044	0.017	0.018	0.049	3.16E-04	0.748	0.018	0.018
Median	-0.002	4.73E-04	0.026	0.022	0.024	0.048	4.99E-04	0.498	0.022	0.025
Mode	-0.002	8.03E-04	0.008	0.028	0.036	0.047	8.68E-04	0.206	0.029	0.037
RAPS	-0.001	3.15E-04	0.042	0.018	0.019	0.048	3.35E-04	0.722	0.018	0.019
cML	-0.001	2.89E-04	0.026	0.017	0.019	0.047	3.26E-04	0.680	0.018	0.020

Average F-statistics when $\theta = 0$ across 500 replicates before and after conditioning are 168 and 168.

Table S7. Simulation results with all valid IVs across 500 replicates. From left to right: causal effect $\theta = 0, 0.05$. The top part corresponds to results from standard MR methods and the bottom part corresponds to results from the CMR implementation. MSE: mean squared error. T1E: type-1 error. SD: standard deviation of estimates. SE: mean of estimated standard error.

	$\theta = 0$					$\theta = 0.05$				
	mean($\hat{\theta}$)	MSE	T1E	SD	SE	mean($\hat{\theta}$)	MSE	Power	SD	SE
					**MR**					
IVW	0.015	2.79E-03	0.042	0.051	0.044	0.064	2.79E-03	0.348	0.051	0.044
Median	-0.001	6.71E-04	0.040	0.026	0.026	0.048	7.21E-04	0.454	0.027	0.027
Mode	-0.004	9.35E-04	0.016	0.030	0.035	0.045	1.00E-03	0.216	0.031	0.036
RAPS	0.001	7.12E-04	0.024	0.027	0.032	0.051	7.35E-04	0.350	0.027	0.033
cML	-0.003	4.10E-04	0.030	0.020	0.022	0.044	5.00E-04	0.440	0.021	0.024
					**CMR**					
IVW	0.002	4.44E-04	0.034	0.021	0.020	0.051	4.61E-04	0.718	0.021	0.021
Median	-0.001	4.76E-04	0.020	0.022	0.025	0.048	5.08E-04	0.482	0.023	0.025
Mode	-0.002	9.33E-04	0.008	0.031	0.037	0.046	9.92E-04	0.210	0.031	0.038
RAPS	0.000	3.22E-04	0.030	0.018	0.020	0.049	3.43E-04	0.698	0.019	0.020
cML	-0.001	2.93E-04	0.020	0.017	0.020	0.047	3.39E-04	0.660	0.018	0.021

Average F-statistics when $\theta = 0$ across 500 replicates before and after conditioning are 168 and 163.

Table S8. Simulation results for Scenario 1 with 20% IVs having LD-induced horizontal pleiotropy and no IV with biological pleiotropy across 500 replicates. From left to right: causal effect $\theta = 0, 0.05$. The top part corresponds to results from standard MR methods and the bottom part corresponds to results from the CMR implementation. MSE: mean squared error. T1E: type-1 error. SD: standard deviation of estimates. SE: mean of estimated standard error.

	$\theta = 0$					$\theta = 0.05$				
	mean($\hat{\theta}$)	MSE	T1E	SD	SE	mean($\hat{\theta}$)	MSE	Power	SD	SE
					**MR**					
IVW	0.032	6.01E-03	0.070	0.071	0.062	0.081	5.99E-03	0.226	0.071	0.062
Median	0.002	9.52E-04	0.058	0.031	0.029	0.051	1.00E-03	0.412	0.032	0.029
Mode	-0.001	1.05E-03	0.032	0.032	0.037	0.048	1.13E-03	0.222	0.034	0.038
RAPS	0.011	2.16E-03	0.044	0.045	0.048	0.061	2.21E-03	0.222	0.046	0.048
cML	-0.004	6.36E-04	0.050	0.025	0.026	0.041	8.35E-04	0.292	0.027	0.028
					**CMR**					
IVW	0.007	6.74E-04	0.018	0.025	0.023	0.056	6.84E-04	0.692	0.025	0.024
Median	0.002	4.90E-04	0.018	0.022	0.026	0.051	5.18E-04	0.504	0.023	0.026
Mode	0.000	9.20E-04	0.012	0.030	0.038	0.049	9.64E-04	0.206	0.031	0.039
RAPS	0.003	3.33E-04	0.022	0.018	0.021	0.052	3.50E-04	0.682	0.019	0.021
cML	0.001	2.85E-04	0.018	0.017	0.020	0.049	3.22E-04	0.650	0.018	0.021

Average F-statistics when $\theta = 0$ across 500 replicates before and after conditioning are 168 and 159.

Table S9. Simulation results for Scenario 1 with 40% IVs having LD-induced horizontal pleiotropy and no IV with biological pleiotropy across 500 replicates. From left to right: causal effect $\theta = 0, 0.05$. The top part corresponds to results from standard MR methods and the bottom part corresponds to results from the CMR implementation. MSE: mean squared error. T1E: type-1 error. SD: standard deviation of estimates. SE: mean of estimated standard error.

	$\theta = 0$					$\theta = 0.05$				
	mean($\hat{\theta}$)	MSE	T1E	SD	SE	mean($\hat{\theta}$)	MSE	Power	SD	SE
					**MR**					
IVW	0.049	9.54E-03	0.078	0.084	0.077	0.099	9.53E-03	0.260	0.084	0.077
Median	0.006	1.90E-03	0.148	0.043	0.032	0.055	1.95E-03	0.444	0.044	0.033
Mode	-0.005	1.72E-03	0.068	0.041	0.039	0.044	1.86E-03	0.228	0.043	0.040
RAPS	0.021	4.52E-03	0.064	0.064	0.063	0.071	4.55E-03	0.218	0.064	0.064
cML	-0.007	1.15E-03	0.072	0.033	0.031	0.034	1.44E-03	0.182	0.035	0.033
					**CMR**					
IVW	0.011	1.07E-03	0.056	0.031	0.026	0.060	1.09E-03	0.622	0.032	0.027
Median	0.003	6.06E-04	0.036	0.025	0.026	0.052	6.28E-04	0.474	0.025	0.027
Mode	0.002	8.91E-04	0.002	0.030	0.038	0.050	9.42E-04	0.216	0.031	0.039
RAPS	0.004	4.95E-04	0.048	0.022	0.022	0.053	5.17E-04	0.642	0.023	0.023
cML	0.001	4.02E-04	0.040	0.020	0.021	0.050	4.46E-04	0.612	0.021	0.022

Average F-statistics when $\theta = 0$ across 500 replicates before and after conditioning are 167 and 153.

Table S10. Simulation results for Scenario 1 with 60% IVs having LD-induced horizontal pleiotropy and no IV with biological pleiotropy across 500 replicates. From left to right: causal effect $\theta = 0, 0.05$. The top part corresponds to results from standard MR methods and the bottom part corresponds to results from the CMR implementation. MSE: mean squared error. T1E: type-1 error. SD: standard deviation of estimates. SE: mean of estimated standard error.

	$\theta = 0$					$\theta = 0.05$				
	mean($\hat{\theta}$)	MSE	T1E	SD	SE	mean($\hat{\theta}$)	MSE	Power	SD	SE
					**MR**					
IVW	0.065	1.35E-02	0.084	0.096	0.089	0.115	1.35E-02	0.240	0.096	0.089
Median	0.008	4.36E-03	0.248	0.066	0.036	0.058	4.43E-03	0.422	0.066	0.036
Mode	-0.010	5.20E-03	0.192	0.072	0.045	0.036	5.35E-03	0.260	0.072	0.046
RAPS	0.035	7.72E-03	0.070	0.081	0.079	0.085	7.72E-03	0.184	0.081	0.079
cML	-0.013	2.14E-03	0.076	0.044	0.040	0.024	2.85E-03	0.104	0.046	0.041
					**CMR**					
IVW	0.015	1.27E-03	0.040	0.033	0.029	0.064	1.27E-03	0.664	0.033	0.029
Median	0.003	6.77E-04	0.038	0.026	0.027	0.052	6.88E-04	0.458	0.026	0.028
Mode	0.000	1.07E-03	0.016	0.033	0.039	0.049	1.12E-03	0.228	0.034	0.040
RAPS	0.005	5.08E-04	0.046	0.022	0.024	0.055	5.21E-04	0.642	0.022	0.024
cML	0.002	4.08E-04	0.034	0.020	0.022	0.050	4.46E-04	0.628	0.021	0.023

Average F-statistics when $\theta = 0$ across 500 replicates before and after conditioning are 167 and 148.

Table S11. Simulation results for Scenario 1 with 80% IVs having LD-induced horizontal pleiotropy and no IV with biological pleiotropy across 500 replicates. From left to right: causal effect $\theta = 0, 0.05$. The top part corresponds to results from standard MR methods and the bottom part corresponds to results from the CMR implementation. MSE: mean squared error. T1E: type-1 error. SD: standard deviation of estimates. SE: mean of estimated standard error.

	$\theta = 0$					$\theta = 0.05$				
	mean($\hat{\theta}$)	MSE	T1E	SD	SE	mean($\hat{\theta}$)	MSE	Power	SD	SE
					**MR**					
IVW	0.051	4.76E-03	0.102	0.046	0.045	0.101	4.73E-03	0.654	0.047	0.045
Median	0.008	7.33E-04	0.064	0.026	0.025	0.057	7.46E-04	0.610	0.026	0.026
Mode	-0.001	8.74E-04	0.018	0.030	0.034	0.047	9.37E-04	0.268	0.031	0.035
RAPS	0.018	1.10E-03	0.030	0.028	0.033	0.068	1.13E-03	0.548	0.028	0.033
cML	0.000	3.68E-04	0.032	0.019	0.020	0.048	4.21E-04	0.626	0.020	0.022
					**CMR**					
IVW	0.037	3.30E-03	0.044	0.044	0.040	0.086	3.26E-03	0.652	0.044	0.040
Median	0.003	4.98E-04	0.018	0.022	0.025	0.052	5.12E-04	0.566	0.023	0.025
Mode	-0.001	7.50E-04	0.004	0.027	0.035	0.048	8.01E-04	0.250	0.028	0.036
RAPS	0.010	6.68E-04	0.016	0.024	0.028	0.060	6.80E-04	0.576	0.024	0.029
cML	0.000	2.94E-04	0.020	0.017	0.019	0.048	3.30E-04	0.698	0.018	0.020

Average F-statistics when $\theta = 0$ across 500 replicates before and after conditioning are 170 and 164.

Table S12. Simulation results for Scenario 2 with 20% IVs having LD-induced horizontal pleiotropy and no IV with biological pleiotropy across 500 replicates. From left to right: causal effect $\theta = 0, 0.05$. The top part corresponds to results from standard MR methods and the bottom part corresponds to results from the CMR implementation. MSE: mean squared error. T1E: type-1 error. SD: standard deviation of estimates. SE: mean of estimated standard error.

	$\theta = 0$					$\theta = 0.05$				
	mean($\hat{\theta}$)	MSE	T1E	SD	SE	mean($\hat{\theta}$)	MSE	Power	SD	SE
					**MR**					
IVW	0.093	1.21E-02	0.266	0.058	0.059	0.143	1.20E-02	0.742	0.059	0.059
Median	0.021	1.53E-03	0.152	0.033	0.027	0.070	1.55E-03	0.690	0.034	0.028
Mode	0.003	1.19E-03	0.044	0.034	0.035	0.052	1.22E-03	0.282	0.035	0.036
RAPS	0.048	4.38E-03	0.106	0.046	0.046	0.098	4.41E-03	0.574	0.046	0.046
cML	0.001	5.09E-04	0.052	0.023	0.022	0.048	5.98E-04	0.522	0.024	0.024
					**CMR**					
IVW	0.071	8.16E-03	0.100	0.056	0.054	0.120	8.07E-03	0.694	0.056	0.054
Median	0.009	6.65E-04	0.050	0.024	0.025	0.058	6.67E-04	0.614	0.025	0.026
Mode	0.001	8.48E-04	0.012	0.029	0.039	0.049	8.66E-04	0.290	0.029	0.040
RAPS	0.026	1.90E-03	0.034	0.035	0.038	0.076	1.90E-03	0.522	0.035	0.038
cML	0.000	3.25E-04	0.032	0.018	0.020	0.048	3.56E-04	0.680	0.019	0.021

Average F-statistics when $\theta = 0$ across 500 replicates before and after conditioning are 172 and 161.

Table S13. Simulation results for Scenario 2 with 40% IVs having LD-induced horizontal pleiotropy and no IV with biological pleiotropy across 500 replicates. From left to right: causal effect $\theta = 0, 0.05$. The top part corresponds to results from standard MR methods and the bottom part corresponds to results from the CMR implementation. MSE: mean squared error. T1E: type-1 error. SD: standard deviation of estimates. SE: mean of estimated standard error.

	$\theta = 0$					$\theta = 0.05$				
	mean($\hat{\theta}$)	MSE	T1E	SD	SE	mean($\hat{\theta}$)	MSE	Power	SD	SE
					**MR**					
IVW	0.146	2.60E-02	0.560	0.068	0.070	0.195	2.58E-02	0.846	0.069	0.071
Median	0.041	3.82E-03	0.304	0.046	0.029	0.091	3.89E-03	0.790	0.047	0.030
Mode	0.009	1.50E-03	0.062	0.038	0.034	0.058	1.56E-03	0.362	0.039	0.035
RAPS	0.095	1.27E-02	0.262	0.061	0.061	0.145	1.28E-02	0.708	0.062	0.061
cML	0.003	6.24E-04	0.058	0.025	0.024	0.049	7.58E-04	0.466	0.028	0.026
					**CMR**					
IVW	0.115	1.79E-02	0.272	0.068	0.066	0.164	1.77E-02	0.802	0.069	0.067
Median	0.016	1.00E-03	0.090	0.027	0.026	0.065	9.85E-04	0.676	0.028	0.027
Mode	0.003	7.31E-04	0.020	0.027	0.038	0.051	7.50E-04	0.358	0.027	0.038
RAPS	0.053	5.39E-03	0.068	0.051	0.050	0.102	5.30E-03	0.536	0.051	0.050
cML	0.002	3.53E-04	0.034	0.019	0.020	0.050	3.92E-04	0.650	0.020	0.021

Average F-statistics when $\theta = 0$ across 500 replicates before and after conditioning are 172 and 157.

Table S14. Simulation results for Scenario 2 with 60% IVs having LD-induced horizontal pleiotropy and no IV with biological pleiotropy across 500 replicates. From left to right: causal effect $\theta = 0, 0.05$. The top part corresponds to results from standard MR methods and the bottom part corresponds to results from the CMR implementation. MSE: mean squared error. T1E: type-1 error. SD: standard deviation of estimates. SE: mean of estimated standard error.

	$\theta = 0$					$\theta = 0.05$				
	mean($\hat{\theta}$)	MSE	T1E	SD	SE	mean($\hat{\theta}$)	MSE	Power	SD	SE
					**MR**					
IVW	0.183	3.87E-02	0.686	0.074	0.076	0.232	3.85E-02	0.942	0.074	0.076
Median	0.066	8.27E-03	0.458	0.063	0.031	0.116	8.37E-03	0.852	0.064	0.032
Mode	0.018	3.05E-03	0.172	0.052	0.035	0.066	3.03E-03	0.444	0.053	0.036
RAPS	0.139	2.45E-02	0.452	0.072	0.071	0.189	2.45E-02	0.826	0.072	0.072
cML	0.002	9.25E-04	0.062	0.030	0.028	0.048	1.14E-03	0.390	0.034	0.029
					**CMR**					
IVW	0.148	2.76E-02	0.450	0.076	0.073	0.197	2.74E-02	0.858	0.077	0.074
Median	0.021	1.35E-03	0.128	0.030	0.027	0.070	1.34E-03	0.712	0.031	0.028
Mode	0.001	7.36E-04	0.034	0.027	0.036	0.049	7.71E-04	0.350	0.028	0.037
RAPS	0.082	1.11E-02	0.152	0.066	0.059	0.131	1.09E-02	0.616	0.066	0.059
cML	0.001	3.94E-04	0.036	0.020	0.021	0.049	4.37E-04	0.616	0.021	0.022

Average F-statistics when $\theta = 0$ across 500 replicates before and after conditioning are 174 and 154.

Table S15. Simulation results for Scenario 2 with 80% IVs having LD-induced horizontal pleiotropy and no IV with biological pleiotropy across 500 replicates. From left to right: causal effect $\theta = 0, 0.05$. The top part corresponds to results from standard MR methods and the bottom part corresponds to results from the CMR implementation. MSE: mean squared error. T1E: type-1 error. SD: standard deviation of estimates. SE: mean of estimated standard error.

	$\theta = 0$					$\theta = 0.1$				
	mean($\hat{\theta}$)	MSE	T1E	SD	SE	mean($\hat{\theta}$)	MSE	Power	SD	SE
					**MR**					
IVW	0.036	0.017	0.046	0.125	0.105	0.127	0.017	0.258	0.127	0.107
Median	0.006	0.005	0.074	0.068	0.061	0.097	0.005	0.318	0.071	0.064
Mode	0.002	0.005	0.022	0.072	0.085	0.087	0.006	0.126	0.078	0.090
RAPS	0.012	0.007	0.044	0.080	0.082	0.104	0.007	0.270	0.082	0.083
cML	-0.002	0.003	0.054	0.056	0.052	0.085	0.004	0.342	0.061	0.056
					**CMR**					
IVW	0.009	0.004	0.040	0.059	0.050	0.100	0.004	0.492	0.063	0.053
Median	0.002	0.003	0.032	0.055	0.057	0.093	0.003	0.324	0.057	0.061
Mode	0.004	0.006	0.004	0.075	0.092	0.089	0.007	0.088	0.080	0.097
RAPS	0.003	0.002	0.044	0.048	0.048	0.096	0.003	0.498	0.051	0.051
cML	-0.001	0.002	0.030	0.045	0.046	0.089	0.002	0.456	0.049	0.049

Average F-statistics when $\theta = 0$ across 500 replicates before and after conditioning are 49 and 47.

Table S16. Simulation results for Scenario 4 with 20% IVs having LD-induced horizontal pleiotropy across 500 replicates. From left to right: causal effect $\theta = 0, 0.1$. The top part corresponds to results from standard MR methods and the bottom part corresponds to results from the CMR implementation. MSE: mean squared error. T1E: type-1 error. SD: standard deviation of estimates. SE: mean of estimated standard error.

	$\theta = 0$					$\theta = 0.1$				
	mean($\hat{\theta}$)	MSE	T1E	SD	SE	mean($\hat{\theta}$)	MSE	Power	SD	SE
					**MR**					
IVW	0.103	0.056	0.112	0.213	0.192	0.194	0.055	0.182	0.214	0.192
Median	0.018	0.019	0.200	0.136	0.078	0.110	0.020	0.344	0.140	0.081
Mode	-0.012	0.013	0.086	0.114	0.094	0.070	0.014	0.132	0.115	0.100
RAPS	0.062	0.037	0.084	0.182	0.167	0.155	0.036	0.172	0.183	0.169
cML	-0.014	0.009	0.092	0.093	0.080	0.063	0.011	0.134	0.097	0.085
					**CMR**					
IVW	0.021	0.007	0.034	0.082	0.067	0.112	0.007	0.354	0.084	0.069
Median	0.002	0.003	0.016	0.057	0.064	0.091	0.004	0.248	0.061	0.067
Mode	-0.002	0.006	0.004	0.079	0.100	0.081	0.007	0.074	0.083	0.104
RAPS	0.005	0.003	0.036	0.053	0.059	0.097	0.003	0.346	0.056	0.061
cML	-0.004	0.002	0.034	0.047	0.051	0.086	0.003	0.352	0.051	0.054

Average F-statistics when $\theta = 0$ across 500 replicates before and after conditioning are 48 and 44.

Table S17. Simulation results for Scenario 4 with 60% IVs having LD-induced horizontal pleiotropy across 500 replicates. From left to right: causal effect $\theta = 0, 0.1$. The top part corresponds to results from standard MR methods and the bottom part corresponds to results from the CMR implementation. MSE: mean squared error. T1E: type-1 error. SD: standard deviation of estimates. SE: mean of estimated standard error.

	$\theta = 0$					$\theta = 0.05$				
	mean($\hat{\theta}$)	MSE	T1E	SD	SE	mean($\hat{\theta}$)	MSE	Power	SD	SE
					**MR**					
IVW	4.18E-05	4.21E-05	0.034	0.006	0.007	0.050	4.84E-05	1	0.007	0.007
Median	-1.65E-05	6.82E-05	0.024	0.008	0.009	0.050	7.35E-05	0.998	0.009	0.010
Mode	-2.40E-04	8.88E-05	0.024	0.009	0.011	0.050	9.49E-05	0.988	0.010	0.011
RAPS	1.45E-04	4.37E-05	0.026	0.007	0.007	0.050	4.62E-05	1	0.007	0.007
cML	-1.82E-04	3.99E-05	0.02	0.006	0.007	0.049	4.54E-05	1	0.007	0.008
					**CMR**					
IVW	4.66E-05	4.21E-05	0.034	0.006	0.007	0.049	4.75E-05	1	0.007	0.007
Median	-1.62E-05	6.82E-05	0.024	0.008	0.009	0.050	7.36E-05	0.998	0.009	0.010
Mode	-2.38E-04	8.89E-05	0.024	0.009	0.011	0.049	9.48E-05	0.988	0.010	0.011
RAPS	1.50E-04	4.37E-05	0.026	0.007	0.007	0.050	4.50E-05	1	0.007	0.007
cML	-1.77E-04	3.99E-05	0.02	0.006	0.007	0.049	4.46E-05	1	0.007	0.008

Table S18. Simulation results for Scenario SA across 500 replicates. From left to right: causal effect $\theta = 0, 0.05$. The top part corresponds to results from standard MR methods and the bottom part corresponds to results from the CMR implementation. MSE: mean squared error. T1E: type-1 error. SD: standard deviation of estimates. SE: mean of estimated standard error.

	$\theta = 0$					$\theta = 0.2$				
	mean($\hat{\theta}$)	MSE	T1E	SD	SE	mean($\hat{\theta}$)	MSE	Power	SD	SE
						**MR**				
IVW	0.046	3.28E-03	0.386	0.035	0.029	0.245	3.29E-03	1	0.036	0.030
Median	0.038	3.07E-03	0.568	0.041	0.014	0.239	3.20E-03	1	0.041	0.016
Mode	0.025	4.37E-03	0.356	0.061	0.023	0.220	3.49E-03	0.994	0.056	0.027
RAPS	0.032	2.16E-03	0.242	0.034	0.028	0.232	2.23E-03	1	0.034	0.029
cML	-0.010	4.54E-04	0.134	0.019	0.017	0.175	1.57E-03	0.992	0.030	0.021
						**CMR**				
IVW	4.22E-03	2.06E-04	0.052	0.014	0.009	0.203	2.61E-04	1	0.016	0.011
Median	5.20E-04	7.45E-05	0.024	0.009	0.010	0.200	1.20E-04	1	0.011	0.012
Mode	-7.44E-05	1.53E-04	0.002	0.012	0.016	0.199	2.07E-04	1	0.014	0.018
RAPS	1.04E-03	5.94E-05	0.038	0.008	0.008	0.200	9.10E-05	1	0.010	0.010
cML	-9.69E-05	4.88E-05	0.028	0.007	0.008	0.198	8.35E-05	1	0.009	0.010

Table S19. Simulation results for Scenario SB across 500 replicates. From left to right: causal effect $\theta = 0, 0.2$. The top part corresponds to results from standard MR methods and the bottom part corresponds to results from the CMR implementation. MSE: mean squared error. T1E: type-1 error. SD: standard deviation of estimates. SE: mean of estimated standard error.

	$\theta = 0$					$\theta = 0.2$				
	mean($\hat{\theta}$)	MSE	T1E	SD	SE	mean($\hat{\theta}$)	MSE	Power	SD	SE
						**MR**				
IVW	1.368	2.561	0.528	0.832	0.693	1.569	2.565	0.62	0.832	0.693
Median	1.641	4.278	0.884	1.260	0.151	1.846	4.304	0.898	1.263	0.153
Mode	0.965	3.309	0.666	1.544	0.180	1.122	3.175	0.684	1.526	0.185
RAPS	1.018	2.748	0.87	1.310	0.079	1.259	2.816	0.838	1.303	0.081
cML	-0.234	0.117	0.308	0.250	0.186	-0.129	0.158	0.16	0.224	0.177
						**CMR**				
IVW	0.069	0.060	0.066	0.234	0.094	0.267	0.059	0.886	0.235	0.095
Median	0.007	0.006	0.044	0.075	0.078	0.205	0.006	0.764	0.076	0.080
Mode	-0.001	0.010	0.012	0.098	0.121	0.197	0.010	0.368	0.098	0.123
RAPS	0.011	0.005	0.052	0.072	0.071	0.210	0.005	0.852	0.072	0.072
cML	-0.003	0.003	0.04	0.055	0.062	0.191	0.003	0.854	0.058	0.065

Table S20. Simulation results for Scenario SC across 500 replicates. From left to right: causal effect $\theta = 0, 0.2$. The top part corresponds to results from standard MR methods and the bottom part corresponds to results from the CMR implementation. MSE: mean squared error. T1E: type-1 error. SD: standard deviation of estimates. SE: mean of estimated standard error.

Supplemental Notes

S1 Nonlinear genetic effects on traits

In the main text Eqs (3)-(4), we introduce the proposed CMR framework assuming linearity of genetic effects of variants Z_j and G_j on the exposure X. As suggested by a reviewer, we will investigate the performance of CMR (and standard MR) when the linearity of the SNP-exposure effect does not hold in this section. First, we consider the following scenario with no horizontal pleiotropy pathway from either Z_j or G_j to Y (Fig. S7A):

$$\begin{aligned} X &= b_{Xj} Z_j + f(G_j) + \epsilon_X, \\ Y &= \theta X + \epsilon_Y, \end{aligned} \tag{1}$$

where ϵ_X and ϵ_Y are correlated random errors due to unmeasured confounding, and independent of variants Z_j and G_j; X, Y, Z_j, G_j are assumed to be standardized, and $f(G_j)$ can be some non-linear function of G_j. In the **working** GWAS association model assuming linearity between a variant and a trait, we have the GWAS marginal estimates of Z_j on the exposure and the outcome as $\hat{\beta}_{Xj} = (Z_j'Z_j)^{-1}(Z_j'X)$ and $\hat{\beta}_{Yj} = (Z_j'Z_j)^{-1}(Z_j'Y)$ respectively. Then we have

$$\beta_{Xj} := \text{plim}\,\hat{\beta}_{Xj} = \text{plim}(Z_j'Z_j)^{-1}Z_j(b_{Xj}Z_j + f(G_j) + \epsilon_X) = b_{Xj} + \mathbb{E}[Z_j f(G_j)], \tag{2}$$

$$\beta_{Yj} := \text{plim}\,\hat{\beta}_{Yj} = \text{plim}(Z_j'Z_j)^{-1}Z_j(\theta X + \epsilon_Y) = \theta\beta_{Xj}. \tag{3}$$

From Eq. (3), we can see that the genetic instrument Z_j is valid with no horizontal pleiotropy in the standard MR framework. The same argument applies to the GWAS marginal estimates of G_j on X and Y, indicating that G_j is also a valid IV in the standard MR framework.

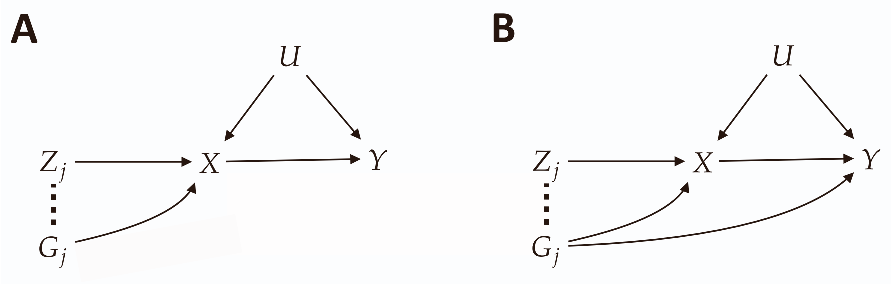

Figure S7. Directed acyclic graph among genetic variants Z_j, G_j, exposure X, outcome Y, and unmeasured confounding U.

In the **working** CMR model, we have the conditional genetic effect estimates of Z_j (conditioning on G_j) on X and Y as $\hat{\beta}^*_{Xj} = \frac{(Z_j - \hat{\rho}_j G_j)'X}{N(1-\hat{\rho}_j^2)}$ and $\hat{\beta}^*_{Yj} = \frac{(Z_j - \hat{\rho}_j G_j)'Y}{N(1-\hat{\rho}_j^2)}$ respectively, where $\hat{\rho}_j = Z_j' G_j / N$. By the same argument on $\beta^*_{Yj} := \text{plim}\, \hat{\beta}^*_{Yj}$ and $\beta^*_{Xj} := \text{plim}\, \hat{\beta}^*_{Xj}$, we have $\beta^*_{Yj} = \text{plim}\, \frac{(Z_j - \hat{\rho}_j G_j)'(\theta X + \epsilon_Y)}{N(1-\hat{\rho}_j^2)} = \theta \beta^*_{Xj}$. Hence, Z_j is also a valid IV in the CMR framework (and so is G_j). As to be shown in the additional simulation studies, both standard MR and CMR approaches performed well when $f(G_j)$ is nonlinear in the absence of pleiotropic pathway.

Next, we consider the scenario where G_j has an additional pleiotropic pathway to Y (Fig. S7B) as follows:

$$\begin{aligned} X &= b_{Xj} Z_j + f(G_j) + \epsilon_X, \\ Y &= \theta X + r_j G_j + \epsilon_Y. \end{aligned} \tag{4}$$

In CMR, we have the conditional genetic effect of Z_j on the outcome $\beta^*_{Yj} := \text{plim}\, \hat{\beta}^*_{Yj}$:

$$\begin{aligned} \beta^*_{Yj} &= \text{plim}\, \frac{1}{N(1-\hat{\rho}_j^2)} (Z_j - \hat{\rho}_j G_j)'(\theta X + r_j G_j + \epsilon_Y) \\ &= \theta \underbrace{\text{plim}\, \frac{1}{N(1-\hat{\rho}_j^2)} (Z_j - \hat{\rho}_j G_j)'X}_{\beta^*_{Xj}} + \frac{r_j}{1-\rho_j^2} \underbrace{\text{plim}\, \frac{1}{N} (Z_j - \hat{\rho}_j G_j)'G_j}_{\rho_j - \rho_j = 0} \qquad (5) \\ &= \theta \beta^*_{Xj}, \qquad (6) \end{aligned}$$

which suggests that Z_j is still **valid** in the CMR framework, even when the effect of G_j on X is nonlinear. However, when the pleiotropic effect of G_j is not linear in Eq. (4), i.e., $Y = \theta X + h(G_j) + \epsilon_Y$, where $h(G_j)$ is some nonlinear function of G_j, then the second term in Eq. (5) is generally non-zero, making Z_j still invalid in CMR. We will consider

both linear and nonlinear pleiotropic effects $h(G_j)$ in the simulation next.

S1.1 Simulation studies with nonlinear genetic effects

We performed simulation studies to investigate the performance of the five robust MR methods discussed in the main text within both the standard MR and CMR frameworks. We followed the same procedure to generate genotype data as in Section 4.4 in the main text, and generated phenotype data from the following model (Fig. S7):

$$X = \sum_{j=1}^{20} b_{Xj} Z_j + \sum_{j=1}^{20} \gamma_j f(G_j) + U + e_X,$$
$$Y = \theta X + \sum_{j=1}^{20} r_j h(G_j) + U + e_Y,$$

where $U, e_X, e_Y \sim \mathcal{N}(0,1)$ independently. We considered the following three scenarios:

SA: For $j = 1, \ldots, 20$, $b_{Xj} \sim$ Uniform(0.1,0.2), $\gamma_j \sim$ Uniform(0.1,0.2), $r_j = 0$, $f(G_j) = \exp(G_j)$.

SB: For $j = 1, \ldots, 20$, $b_{Xj} = 0.4$, $\gamma_j = 0.2$, $r_j = 0.2$, $f(G_j) = G_j^2$, $h(G_j) = G_j$.

SC: For $j = 1, \ldots, 20$, $b_{Xj} = 0.4$, $\gamma_j = 0.2$, $r_j = 0.2$, $f(G_j) = G_j^2$, $h(G_j) = \exp(2 * G_j)$.

Briefly, in all three scenarios, the effect of G_j on X was nonlinear, and Z_j did not have biological pleiotropy on Y. In Scenario SA, G_j did not have a pleiotropic pathway on Y; in Scenario SB, the (biological) pleiotropy effect of G_j on Y was linear; and in Scenario SC, the pleiotropy effect of G_j on Y was nonlinear.

In total, 500 replicates were generated in each scenario. To mimic a two-sample setup, we randomly selected two independent sets of 50000 individuals, and calculated GWAS summary data for the exposure and the outcome respectively. We used all available unrelated white-ancestry UKB individuals as the LD reference panel. For Scenario SA, we selected independent IVs (GWAS p-value $< 5 \times 10^{-8}$) after LD clumping in each block.

For Scenarios SB and SC, we selected Z_j as IVs to investigate the 'oracle' performance of CMR. Then we applied standard MR and CMR methods with the selected IVs.

Simulation results are provided in Tables S18 to S20. First, in Scenario SA (Table S18), although we used clumping to select IVs, which means both Z_j and G_j might be selected, all MR methods performed well in both the standard MR and CMR frameworks because both Z_j and G_j were valid instruments (as discussed in Section S1). Furthermore, MR and CMR methods gave generally similar results, as COJO oftentimes did not identify any outcome-associated SNPs. In Scenarios SB and SC, all standard MR methods did not perform well due to the LD-induced pleiotropy from G_j. After adjusting for LD-induced pleiotropy, CMR methods had different degrees of improvement over their corresponding MR counterparts. In particular, in Scenario SB (Table S19) with linear pleiotropic effect, all CMR methods performed well, aligned with our previous discussion (Eq. (6)). However, in Scenario SC (Table S20) with nonlinear pleiotropic effect $h(G_j)$, CMR failed to *fully* remove LD-induced pleiotropy, and CMR-IVW yielded more biased results than other robust methods.
